# Bacterial extracellular vesicles and direct microbial contact drive species-specific corneal epithelial reprogramming

**DOI:** 10.3389/fcimb.2026.1899147

**Published:** 2026-08-12

**Authors:** Noelia Blanco-Agudín, Suhui Ye, Sara González-Fernández, Iván Fernández-Vega, Jesús Merayo-Lloves, Luis M. Quirós

**Affiliations:** 1 Instituto Universitario Fernández-Vega (IUFV), Fundación de Investigación Oftalmológica, Oviedo, Spain; 2 Departamento de Biología Funcional, University of Oviedo, Oviedo, Spain; 3 Instituto de Investigación Sanitaria del Principado de Asturias (ISPA), Oviedo, Spain; 4 Department of Pathology, Hospital Universitario Central de Asturias (HUCA), Oviedo, Spain; 5 Biobank of Principality of Asturias, Oviedo, Spain

**Keywords:** bacterial extracellular vesicles, corneal epithelium, exosomes, host-microbe interaction, *Pseudomonas aeruginosa*, *Staphylococcus epidermidis*

## Abstract

**Background:**

Extracellular vesicles (EVs) are important mediators of host–microorganism communication at epithelial surfaces. However, their role in shaping corneal epithelial responses to ocular-associated bacteria remains poorly understood. Here, we investigated the species-specific effects of *Pseudomonas aeruginosa* and *Staphylococcus epidermidis* on human corneal epithelial cells, focusing on bacterial extracellular vesicles (BEVs), direct microbial contact, and epithelial exosomal remodeling.

**Methods:**

Human corneal epithelial cells were exposed to BEVs or co-cultured with *P. aeruginosa* or *S. epidermidis*. Transcriptomic, proteomic, and extracellular vesicle RNA analyses were performed together with functional assays evaluating epithelial metabolic activity, motility, and apoptosis.

**Results:**

BEVs from *P. aeruginosa* induced a strong pro-inflammatory epithelial response associated with IL-17, TNF, and NF-κB signaling, extracellular matrix remodeling, and increased cell motility. Direct bacterial contact further amplified these effects, triggering extensive transcriptional reprogramming and remodeling of epithelial exosomal cargo, including enrichment of proteasome-associated proteins and apoptosis-related microRNAs. Functional apoptosis assays confirmed that exosomes released following *P. aeruginosa* exposure acquired a modest but significant pro-apoptotic activity. In contrast, *S. epidermidis* induced a substantially milder transcriptional response characterized by limited differential gene expression, preferential detection of regulatory non-coding RNAs, and distinct exosomal remodeling associated with cell cycle and signaling pathways. Notably, responses induced by BEVs and direct bacterial contact showed limited overlap, indicating complementary mechanisms of epithelial regulation.

**Conclusions:**

Together, these findings demonstrate that BEVs and direct microbial contact act as complementary and species-specific drivers of corneal epithelial reprogramming. Our results further support a model in which BEVs function as early signaling mediators, whereas epithelial-derived exosomes contribute to the propagation and modulation of these responses across the corneal epithelium.

## 1 Introduction

Host-associated microbiota contributes to epithelial homeostasis, immune regulation, and tissue repair across mucosal surfaces (Belkaid and Hand, 2014; Thomas et al., 2017). At the same time, these microbial communities include opportunistic species capable of driving pathological responses under dysbiotic conditions, highlighting the importance of understanding host–microorganism communication mechanisms.

The corneal epithelium represents a specialized mucosal interface continuously exposed to environmental and microbial challenges while maintaining optical transparency, immune balance, and regenerative capacity (Eghrari et al., 2015). The ocular surface harbors a low-biomass, paucibacterial microbial community whose functional relevance is increasingly recognized in both epithelial homeostasis and inflammatory processes (Doan et al., 2016; Teweldemedhin et al., 2017; Ozkan and Willcox, 2019; Andersson et al., 2021; Das et al., 2022). Among ocular-associated microorganisms, *Staphylococcus epidermidis* is generally considered a low-virulence commensal associated with ocular surface equilibrium (Otto, 2009; Ozkan and Willcox, 2019), whereas *Pseudomonas aeruginosa* is a major opportunistic pathogen responsible for severe microbial keratitis (Fleiszig et al., 2020). These two species were selected to represent biologically relevant commensal and pathogenic interactions at the ocular surface, allowing comparison of extracellular vesicle-mediated communication under physiological and disease-associated conditions. However, the mechanisms by which different ocular-associated bacterial species modulate corneal epithelial responses remain poorly understood.

Extracellular vesicle (EV)-mediated communication has emerged as a conserved mechanism of intercellular signaling across biological systems (Margutti et al., 2024). Both eukaryotic and prokaryotic cells release EVs capable of transferring proteins, lipids, and nucleic acids between organisms and tissues. In bacteria, extracellular vesicles (bacterial extracellular vesicles, BEVs) contribute to bacterial adaptation, virulence, and host interaction through the delivery of bioactive cargo (Toyofuku et al., 2019; Moghaddam et al., 2025). In parallel, host-derived EVs, including exosomes, participate in immune regulation and tissue communication and can be remodeled during infection and inflammatory processes (Kalluri and LeBleu, 2020; Blanco-Agudín et al., 2025). Increasing evidence indicates that EVs mediate bidirectional communication between microorganisms and host tissues, shaping epithelial responses under both physiological and pathological conditions (Caruana and Walper, 2020; Margutti et al., 2024). As interest in EV-based diagnostics and therapeutics continues to grow, increasing attention has also been devoted to the impact of isolation and characterization strategies on EV purity, cargo composition, and biological interpretation, particularly in ocular applications where efficient delivery and standardized methodologies remain major challenges (Verma et al., 2025b).

Here, we investigated the species-specific effects of *P. aeruginosa* and *S. epidermidis* on human corneal epithelial cells by integrating BEV-mediated signaling, direct microbial contact, and epithelial exosomal remodeling. Our results reveal complementary but distinct mechanisms of epithelial reprogramming induced by pathogenic and commensal-associated bacteria, highlighting EVs as important mediators of host–microorganism communication at the ocular surface.

## 2 Materials and methods

### 2.1 Cell lines, bacterial strains and culture conditions

The immortalized human corneal epithelial cell line HCE-2 [50.B1] (ATCC CRL-11135; American Type Culture Collection, Manassas, VA, USA) was used in this study. Cells were cultured in Dulbecco’s modified Eagle’s medium/Ham’s F-12 (DMEM/F-12) supplemented with fetal bovine serum, insulin, and epidermal growth factor, and maintained at 37 °C in a humidified atmosphere containing 5% CO_2_, as previously described (García et al., 2016).

*Pseudomonas aeruginosa* and *Staphylococcus epidermidis* strains were obtained from the Hospital Universitario Central de Asturias (Oviedo, Spain). Bacteria were routinely cultured in brain–heart infusion (BHI) medium at 37 °C under agitation until mid-exponential phase (OD_600_ ≈ 0.5) prior to experimental use.

### 2.2 Isolation and purification of HCE-2–derived exosomes

For the isolation of HCE-2–derived exosomes (Exo-HCE), cells were cultured to ~80% confluence, washed with phosphate-buffered saline (PBS), and incubated for 24 h in DMEM/F-12 supplemented with exosome-depleted fetal bovine serum (FBS; Gibco, Thermo Fisher Scientific, Waltham, MA, USA) and gentamicin at 0.5 μg/mL. The gentamicin concentration (0.5 μg/mL) was selected to mirror the antibiotic conditions used in the *S. epidermidis* co-culture model, thereby providing a basal epithelial exosome control under comparable antibiotic exposure. Conditioned media were collected and concentrated by ultrafiltration using 100 kDa molecular weight cut-off (MWCO) polyethersulfone (PES) concentrator tubes (Pierce™, Thermo Fisher Scientific).

Exosomes were isolated using the Total Exosome Isolation from Cell Culture Media kit (Invitrogen™, Thermo Fisher Scientific) according to the manufacturer’s instructions and further purified by size-exclusion chromatography using EX03 Exo-spin™ mini columns (Cell Guidance Systems, Cambridge, UK). This combined approach was used to improve vesicle purity and reduce contamination from non-vesicular components.

This isolation and characterization strategy is consistent with previous studies on primary human limbal epithelial cell-derived exosomes, which reported comparable vesicle size distributions and demonstrated that alterations in exosomal cargo were associated with functional effects in recipient epithelial cells (Verma et al., 2023).

Exosomes were eluted in PBS and quantified as previously described (Lozano et al., 2022).

### 2.3 Purification of bacterial extracellular vesicles

BEVs were isolated by adapting previously described protocols (Bos et al., 2021; Ito et al., 2021). Briefly, bacterial cultures were grown to mid-exponential phase (OD_600_ ≈ 0.5), diluted 1:5 in 250 mL DMEM/F-12/exo_aa_free, and incubated for 24 h at 37 °C in 5% CO_2_ to allow vesicle release under defined conditions.

Culture media were sequentially centrifuged at 4,500 × g for 20 min and 12,800 × g for 20 min at 4 °C to remove cells and debris. Supernatants were filtered through 0.22 μm filters (Sigma-Aldrich, Merck, Darmstadt, Germany), and sterility was confirmed by plating 100 μL of the final filtrate onto brain–heart infusion (BHI) agar plates, followed by incubation at 37 °C for 48 h, during which no bacterial growth was detected.

Filtered media were concentrated by ultrafiltration using 100 kDa molecular weight cut-off (MWCO) polyethersulfone (PES) concentrator tubes (Pierce™, Thermo Fisher Scientific) and subjected to ultracentrifugation at 100,000 × g for 2.5 h at 4 °C using an SW 60 Ti swinging-bucket rotor (Beckman Coulter, Brea, CA, USA; k-factor = 45). Pellets were washed in phosphate-buffered saline (PBS) and ultracentrifuged again under the same conditions to reduce contamination from soluble proteins and non-vesicular components.

The final BEV pellets were resuspended in PBS. Total protein content was determined by BCA assay for batch characterization, and BEV concentration was determined by nanoparticle tracking analysis (NTA). The representative particle-to-protein ratios were 1.1 × 10^10^ particles/μg protein for *P. aeruginosa* BEVs and 1.5 × 10^9^ particles/μg protein for *S. epidermidis* BEVs. All biological experiments were normalized according to particle number (BEVs/cell), whereas protein measurements were used exclusively for batch characterization and downstream analytical workflows.

### 2.4 Isolation of exosomes from corneal epithelial–bacterial co-cultures

Bacterial cultures grown in DMEM/F-12/exo_aa_free were adjusted to OD_600_ = 0.5. HCE-2 cells were cultured to ~80% confluence in DMEM/F-12, washed with PBS, and incubated with four volumes of DMEM/F-12/exo_aa_free and one volume of bacterial suspension for 1 h at 37 °C in 5% CO_2_.

Following the initial adhesion period, cultures were washed to remove non-adherent bacteria, whereas bacteria attached to the epithelial monolayer remained associated with the cells throughout the experiment. Gentamicin was subsequently added at the minimum inhibitory concentration (MIC) required for each bacterial species (6.25 μg/mL for *P. aeruginosa*; 0.5 μg/mL for *S. epidermidis*) to prevent exponential bacterial proliferation rather than to eliminate the bacteria remaining attached to the epithelial monolayer. This approach maintained a stable bacterial burden, allowed sustained host–bacterium interaction, and preserved epithelial cell viability during the prolonged co-culture period. After 4 h, the medium was replaced with fresh DMEM/F-12/exo_aa_free containing the corresponding gentamicin concentration, and cultures were maintained for an additional 24 h. Owing to the higher growth rate and cytotoxicity of *P. aeruginosa*, conditioned media were collected and replaced every 8 h using fresh medium containing the same gentamicin concentration to minimize epithelial damage and maintain stable co-culture conditions.

Conditioned media were sequentially centrifuged (2,000 × g, 30 min; 12,800 × g, 20 min; 4 °C) and filtered. Samples were concentrated using 100 kDa MWCO concentrator tubes. Exosomes were separated from BEVs by two sequential rounds of the Total Exosome Isolation from Cell Culture Media reagent, followed by purification using EX03 Exo-spin™ mini columns.

Purified exosomes were resuspended in PBS and stored at −20 °C until use.

### 2.5 Characterization of EVs by nanoparticle tracking analysis and transmission electron microscopy

EV size distribution, concentration, and morphology were determined by NTA and transmission electron microscopy (TEM) as previously described (Lozano et al., 2022). For NTA, EV samples were diluted in particle-free phosphate-buffered saline (PBS) to achieve optimal particle concentration and analyzed using a NanoSight LM10 instrument equipped with a 405 nm laser (Malvern Panalytical, Malvern, UK). Videos were acquired using a camera level of 15 and a detection threshold of 5. Particle size and concentration were calculated using the NTA software under standardized acquisition conditions.

For TEM analysis, EV suspensions were applied onto formvar/carbon-coated grids, negatively stained with phosphotungstic acid, and examined using a transmission electron microscope to assess vesicle morphology and size distribution.

### 2.6 Western blot analysis

Proteins were separated on 4–20% ProteinEle^®^ Precast Tris–Glycine gels (TransGen Biotech, Beijing, China) and transferred to nitrocellulose membranes. Membranes were blocked with 5% skim milk for 1 h and incubated overnight at 4 °C with the following primary antibodies: rabbit anti-TSG101 polyclonal antibody (PA5-810949; 1:500), used as an exosomal marker; rabbit anti-*Pseudomonas aeruginosa* OprF polyclonal antibody (PA5-117553; 1:625), used as a BEV-associated marker; and rabbit anti-*Staphylococcus epidermidis* SepA polyclonal antibody (CSB-PA313815ZA01SLD; 1:500), used as a species-specific BEV marker (Invitrogen; Cusabio Biotech, Wuhan, China).

Membranes were then incubated for 1 h at room temperature with mouse anti-rabbit IgG-HRP (sc-2357; Santa Cruz Biotechnology, Dallas, TX, USA). Signals were detected using Immobilon™ Western Chemiluminescent HRP Substrate (MilliporeSigma, Merck, Darmstadt, Germany) and visualized with a UVP imaging system.

### 2.7 Proteomic analysis of EVs by liquid chromatography–tandem mass spectrometry

Lyophilized proteins (40 μg) were resuspended in 0.2% RapiGest (Waters, Milford, MA, USA) in 50 mM ammonium bicarbonate and precipitated overnight with acetone at −20 °C. Pellets were resuspended, and protein concentration was determined using a Qubit microfluorimeter (Invitrogen). Proteins were reduced with dithiothreitol (DTT), alkylated with iodoacetamide, and digested with trypsin (Promega, Madison, WI, USA) at an enzyme-to-protein ratio of 1:40. Digestion was performed in two steps (2 h and overnight at 37 °C) and stopped with 10% trifluoroacetic acid (TFA).

Peptide mixtures were analyzed by LC–MS/MS using an Orbitrap Exploris 480 mass spectrometer coupled to a Vanquish Neo HPLC system (Thermo Fisher Scientific). Samples were separated on an IonOpticks Aurora Elite column using a linear gradient and analyzed in data-independent acquisition (DIA) mode.

Raw data were processed using Spectronaut v19.0 (Biognosys AG, Zurich, Switzerland) in library-free mode against the SwissProt *Homo sapiens* database (UniProt), as the objective of this analysis was to characterize host-derived exosomal cargo. Potential contamination by BEVs was assessed independently by Western blot detection of the BEV markers OprF and SepA, together with exosomal marker profiling, NTA, and TEM, as described in the Results. Protein identification and quantification were performed based on peptide-level data, followed by intensity normalization. Peptide and protein identifications were filtered using a 1% false discovery rate (FDR), as implemented in Spectronaut.

To further assess the identity of the isolated exosomes, the LC-MS/MS proteomic dataset was compared with the proposed core exosomal and exclusion marker datasets reported by Kugeratski et al. (2021). The complete comparison is provided in **Supplementary Table 1**.

### 2.8 EV RNA sequencing by next-generation sequencing

Non-coding RNA (ncRNA) was isolated from 30 μg of EVs using the Exosomal RNA Isolation Kit (Norgen Biotek Corp., Thorold, ON, Canada) and stored at −80 °C until analysis. Following 3′ and 5′ adapter ligation, cDNA was synthesized by reverse transcription and amplified by PCR, with size selection of fragments ranging from 18 to 40 nucleotides.

Library quality and concentration were assessed using a Qubit™ Fluorometer (Thermo Fisher Scientific) and an Agilent 2100 Bioanalyzer (Agilent Technologies, Santa Clara, CA, USA). Sequencing was performed on an Illumina NovaSeq 6000 platform (Illumina, San Diego, CA, USA) using single-end 50 bp reads.

Raw sequencing data (FASTQ files) were processed using fastp to remove adapter sequences, low-quality reads, and contaminants. Clean reads were classified into small RNA biotypes and aligned to the miRBase v22.0 database. Expression levels were normalized as transcripts per million (TPM) and used for downstream analyses.

### 2.9 MTT metabolic activity assay

Cellular metabolic activity was evaluated using the MTT colorimetric assay (CellTiter 96^®^ Non-Radioactive Cell Proliferation Assay, Promega). Although originally developed as a proliferation assay, the method measures the metabolic activity of viable cells and therefore provides an indirect estimate of changes in cell number. Briefly, 1,500 HCE-2 cells per well were seeded in 96-well plates. After 2 h of adhesion, cells were treated with exosomes or bacterial extracellular vesicles (BEVs) at the indicated concentrations. Following 48 or 72 h of incubation, absorbance was measured at 570 nm using a BioTek PowerWave XS microplate reader (BioTek Instruments, Winooski, VT, USA). The assay was performed using five independent biological replicates, each corresponding to an independent culture plate.

To evaluate the potential contribution of gentamicin to epithelial metabolic activity, an additional control experiment was performed in which HCE-2 cells were exposed to gentamicin alone at the highest concentration used during the co-culture experiments (6.25 μg/mL).

### 2.10 Cell motility assays

Cell motility was assessed using a wound-healing assay with Culture-Insert 2 Well devices in µ-Dish 35 mm plates (Ibidi GmbH, Gräfelfing, Germany), generating a 500 μm gap. After reaching 100% confluence, inserts were removed and 400 μL DMEM/F-12/exo_aa_free medium was added. Cells were treated with 10^6^ BEVs per cell or 10^5^ exosomes per cell. Images were captured at 0, 2, 4 and 6 h using an inverted microscope. The assay was performed using three independent biological replicates, each corresponding to an independent culture plate. Wound closure was quantified using ImageJ with identical analysis parameters applied to all images. Image analysis was not performed in a blinded manner.

### 2.11 Annexin V/SYTOX green apoptosis assay

To evaluate whether exosomes derived from bacteria-exposed corneal epithelial cells induced apoptosis, HCE-2 cells were seeded in 24-well plates and cultured until reaching approximately 10^5^ cells per well. Cells were then incubated for 24 h with Exo-HCE or exosomes derived from *P. aeruginosa*-exposed HCE-2 cells (Exo-CoPa) at a concentration of 10^5^ exosomes per cell. Untreated cells served as negative controls, whereas cells treated with 5 μM doxorubicin were used as positive controls.

Following treatment, both adherent and floating cells were collected, centrifuged, and resuspended in PBS/HBSS. Apoptosis was evaluated using the Annexin V Ready Flow Conjugates for Apoptosis Detection Kit (Invitrogen, R37176) together with SYTOX™ Green nucleic acid stain (1 μM; Invitrogen, S7020), according to the manufacturer’s recommendations. Samples were incubated for 15 min at room temperature in the dark and analyzed immediately using a CytoFLEX S flow cytometer (Beckman Coulter). A total of 15,000 events were acquired per sample and analyzed using CytExpert software.

Cells were classified as viable (Annexin V−/SYTOX Green−), early apoptotic (Annexin V+/SYTOX Green−), late apoptotic (Annexin V+/SYTOX Green+), or necrotic (Annexin V−/SYTOX Green+). Three independent biological replicates were analyzed.

### 2.12 Transcriptomic analysis

Total RNA was extracted using the RNeasy Mini Kit with RNase-free DNase treatment (Qiagen, Venlo, The Netherlands). RNA quantity and integrity were assessed using a NanoDrop One spectrophotometer (Thermo Fisher Scientific) and an Agilent 2100 Bioanalyzer (Agilent Technologies). Polyadenylated RNA was isolated from total RNA using poly-T oligo-attached magnetic beads to selectively capture protein-coding transcripts together with polyadenylated long non-coding RNAs (lncRNAs) and antisense transcripts. RNA was subsequently fragmented and reverse-transcribed using random primers, incorporating dUTP during second-strand synthesis.

Libraries were validated by Qubit quantification, qPCR, and Bioanalyzer analysis, and sequenced on an Illumina NovaSeq X Plus platform (Illumina, San Diego, CA, USA). Sequencing quality metrics are summarized in **Supplementary Table 2**. Raw reads were processed using fastp for adapter trimming and quality filtering. Clean reads were aligned to the human reference genome using HISAT2 v2.0.5, and transcript annotation was performed using the Ensembl gene annotation files provided within the Novogene RNA-seq analysis pipeline. Gene-level read counts were generated using featureCounts (v1.5.0-p3). Differential expression analysis was performed using DESeq2 based on raw read counts, whereas FPKM values were calculated for transcript abundance estimation and downstream visualization.

### 2.13 Bioinformatic and statistical analysis

Identified proteins were subjected to Gene Ontology (GO) and Kyoto Encyclopedia of Genes and Genomes (KEGG) enrichment analyses using ShinyGO 0.82 (Ge et al., 2020). Protein–protein interaction networks were analyzed using STRING (Szklarczyk et al., 2021). miRNA target genes were identified using miRTarBase (Huang et al., 2020), considering only targets supported by strong experimental evidence.

Statistical analyses were performed using Statistica (StatSoft) and R (R Core Team, 2023). Functional assays were performed using at least three independent biological replicates. Differences between experimental conditions and controls were assessed using the non-parametric Mann–Whitney U test.

Differential transcriptomic analysis was performed using DESeq2, which applies a generalized linear model based on the negative binomial distribution. Genes were considered differentially expressed when they met the thresholds of adjusted p < 0.05 (Benjamini–Hochberg false discovery rate, FDR) and |log_2_FC| > 1. Differential proteomic and EV miRNA analyses were performed using thresholds of p < 0.05 and |log_2_FC| > 1. Functional enrichment analyses (GO, KEGG, Reactome, Disease Ontology, DisGeNET and GSEA) were based on FDR-adjusted significance values.

## 3 Results

### 3.1 Purification and characterization of HCE-derived exosomes and BEVs

**Figure 1:**
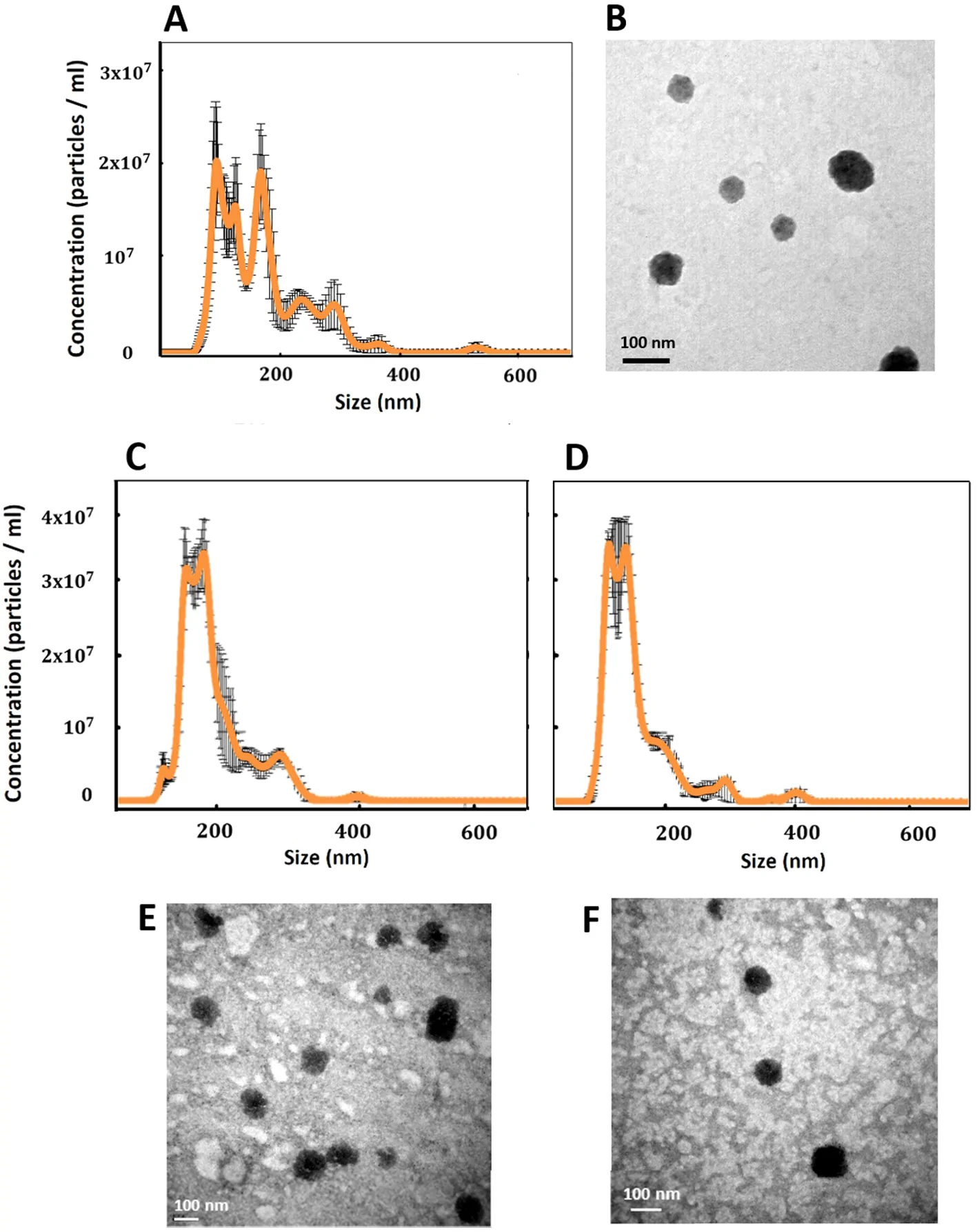
Characterization of extracellular vesicles. Nanoparticle tracking analysis (NTA) size-distribution profiles and transmission electron microscopy (TEM) images of extracellular vesicles isolated in this study. **(A)** Size distribution of HCE-2–derived exosomes (Exo-HCE) and **(B)** representative TEM image. NTA size-distribution profiles of bacterial extracellular vesicles from **(C)** *Pseudomonas aeruginosa* (BEV-Pa) and **(D)** *Staphylococcus epidermidis* (BEV-Se). Representative TEM images of **(E)** BEV-Pa and **(F)** BEV-Se. Scale bars are indicated in each image.

Exo-HCE exhibited a characteristic size distribution, with a main peak at 91 nm and a secondary peak at 163 nm, consistent with the expected size range of exosomal populations (**Figure 1A**). Representative Exo-HCE preparations showed a particle concentration of approximately 3.16 × 10^9^ particles/mL. TEM confirmed the presence of homogeneous, round vesicles with morphology typical of extracellular vesicles (**Figure 1B**).

BEVs displayed species-dependent size distributions. BEVs derived from *P. aeruginosa* (BEV-Pa) showed two closely spaced peaks at 124 and 148 nm and a representative particle concentration of 3.81 × 10^10^ particles/mL (**Figure 1C**), whereas those from *S. epidermidis* (BEV-Se) exhibited peaks at 95 and 124 nm with a representative concentration of 2.09 × 10¹¹ particles/mL (**Figure 1D**). In both cases, TEM analysis confirmed vesicles with morphology and size distributions consistent with previously described BEVs (**Figures 1E, F**).

### 3.2 *Pseudomonas aeruginosa* and *Staphylococcus epidermidis* BEVs differentially modulate corneal epithelial cell behavior

**Figure 2:**
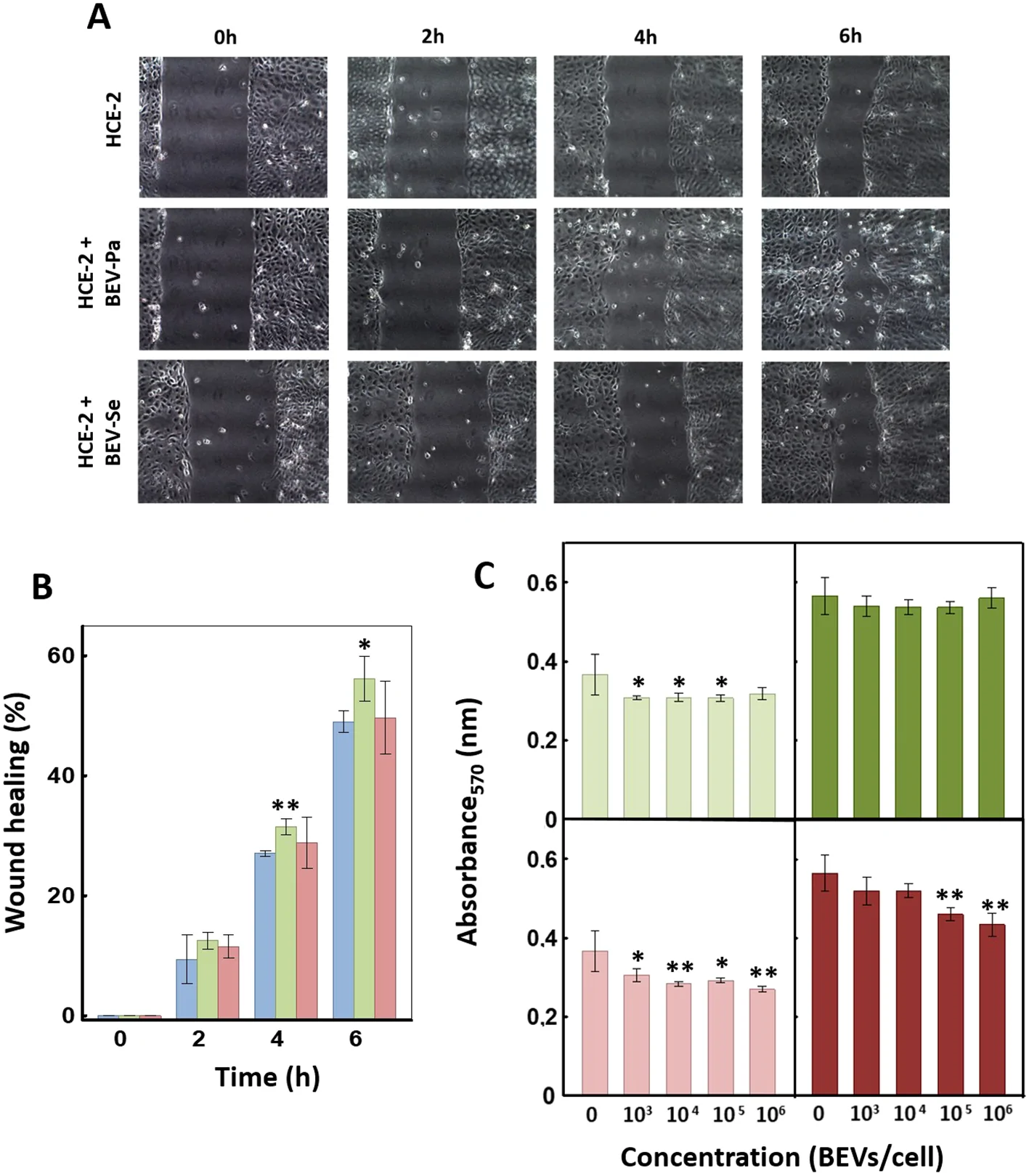
Functional effects of bacterial extracellular vesicles on corneal epithelial cells. **(A)** Representative micrographs from wound-healing assays showing HCE-2 cell monolayers treated with BEVs derived from *Pseudomonas aeruginosa* (BEV-Pa) or *Staphylococcus epidermidis* (BEV-Se). **(B)** Quantification of wound closure, expressed as percentage gap reduction over time in control cells and cells treated with BEV-Pa or BEV-Se. Data are presented as mean ± SD from n = 3 independent biological replicates. **(C)** Cellular metabolic activity assessed by MTT assay at different vesicle-to-cell ratios. Upper panels: BEV-Pa treatment at 48 h and 72 h. Lower panels: BEV-Se treatment at 48 h and 72 h. Data are presented as mean ± standard deviation. Data are presented as mean ± SD from five wells per experimental condition. Statistical significance was assessed using the Mann–Whitney U test. *p* < 0.05 (*) and *p* < 0.01 (**).

The functional effects of BEVs on corneal epithelial cells were first evaluated by wound-healing assays, with closure quantified as percentage gap reduction (**Figure 2A**). BEV-Pa significantly increased cell motility, with closure reaching 31.5% versus 27% in controls at 4 h, and 56% versus 49% at 6 h. In contrast, BEV-Se did not significantly affect gap closure (**Figure 2B**).

Cellular metabolic activity, assessed by MTT assay at different vesicle-to-cell ratios, showed distinct effects depending on vesicle origin. BEV-Pa induced a moderate reduction in metabolic activity at 48 h (~16% versus control across concentrations), which was not maintained at 72 h. At the highest dose (10^6^ BEVs/cell), the effect was not statistically significant. In contrast, BEV-Se induced a stronger inhibitory effect on metabolic activity at 48 h across all doses (−16% to −26.5%), which persisted at 72 h, although slightly attenuated (−18% and −23% at 10^5^ and 10^6^ BEVs/cell, respectively) (**Figure 2C**).

Transcriptomic analysis revealed that these functional differences were associated with distinct molecular responses. A total of 12,167, 12,155, and 12,230 transcripts were detected in control, BEV-Pa-treated, and BEV-Se-treated cells, respectively, with 11,725 transcripts shared across conditions. Venn diagram analysis indicated a largely conserved transcriptome, with regulation restricted to a subset of genes. Heatmap analysis revealed distinct expression patterns depending on vesicle origin, indicating species-specific epithelial responses (**Supplementary Figure 1**).

**Figure 3:**
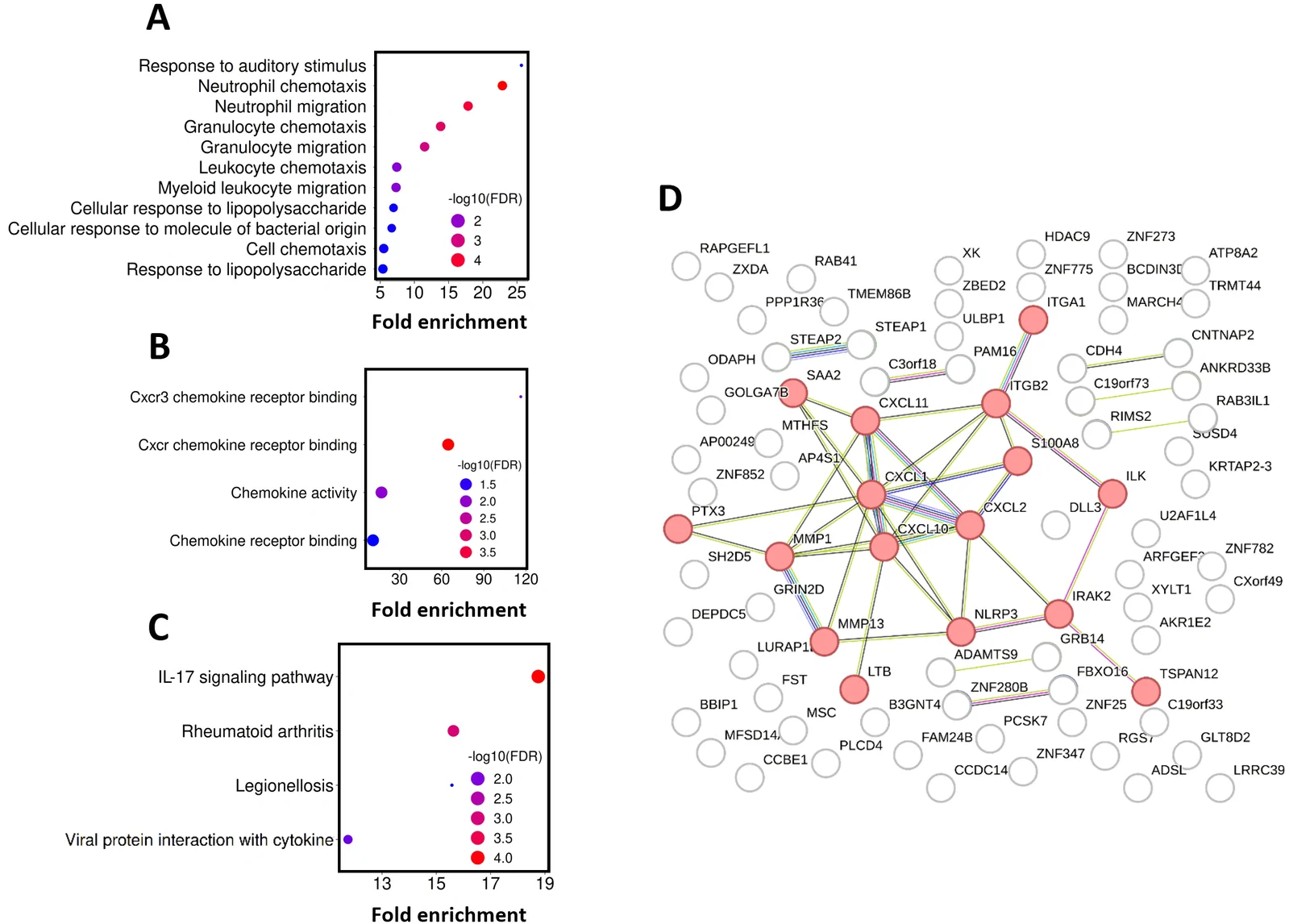
Functional enrichment analysis of genes expressed *de novo* in HCE-2 cells following exposure to BEV-Pa. Gene Ontology (GO) enrichment analysis of genes expressed *de novo* (i.e., not detected in control conditions) in HCE-2 cells following exposure to BEV-Pa, showing **(A)** biological processes and **(B)** molecular functions. **(C)** Kyoto Encyclopedia of Genes and Genomes (KEGG) pathway enrichment analysis. In dot plots, dot size represents the number of genes associated with each term, and color indicates the adjusted statistical significance (Benjamini–Hochberg FDR). Differential gene expression was calculated from n = 3 independent biological replicates using DESeq2 (negative binomial generalized linear model). **(D)** Functional and physical interaction network predicted using the STRING database. A highly interconnected cluster of genes associated with neutrophil chemotaxis and IL-17 signaling is highlighted (p = 1 × 10^-16^).

BEV-Pa induced *de novo* expression of 186 genes and triggered a robust pro-inflammatory transcriptional program. Gene Ontology (GO) enrichment analysis highlighted innate immune activation and inflammatory responses, including neutrophil and granulocyte chemotaxis and responses to bacterial components. Consistently, KEGG pathway analysis revealed strong enrichment in IL-17 signaling (enrichment ratio: 18.7; FDR: 8.2 × 10^-5^) (**Figures 3A–C**). STRING network analysis identified a highly interconnected cluster of 16 genes (p = 1 × 10^-16^) associated with inflammation, chemotaxis, and extracellular matrix (ECM) remodeling (**Figure 3D**), including adhesion and integrin-related genes (*ITGA1*, *ITGB2*, *ILK*), inflammatory mediators (*SAA2*, *PTX3*, *LTB*, *NLRP3*, *IRAK2*), chemokines (*CXCL1*, *CXCL2*, *CXCL10*, *CXCL11*) and their receptor *CXCR3*, as well as ECM-degrading enzymes (*MMP1*, *MMP2*).

**Figure 4:**
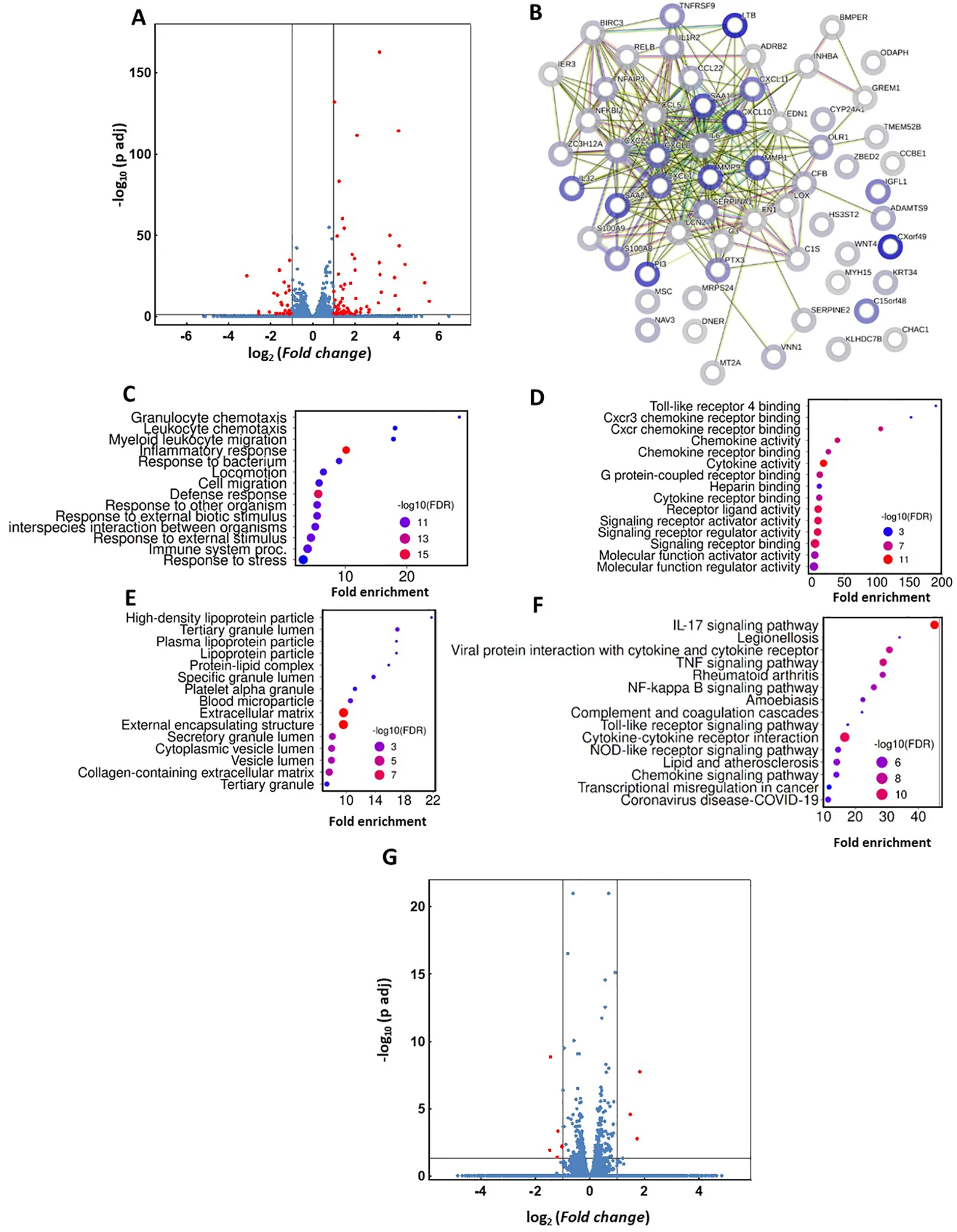
Differential gene expression and functional enrichment analysis in HCE-2 cells following exposure to BEV-Pa and BEV-Se. **(A)** Volcano plot showing differential gene expression in HCE-2 cells following exposure to BEV-Pa relative to control conditions. Each dot represents a gene; red dots indicate significantly upregulated genes (adjusted *p* < 0.05 and |log_2_FC| > 1). Differential expression was calculated from n = 3 independent biological replicates using DESeq2 (negative binomial generalized linear model), and *p*-values were adjusted using the Benjamini–Hochberg false discovery rate (FDR) correction. **(B)** Protein–protein interaction network of the corresponding upregulated gene products, predicted using the STRING database. The intensity of the blue halo reflects the degree of overexpression relative to control conditions. Gene Ontology (GO) enrichment analysis of upregulated genes following BEV-Pa exposure, showing **(C)** biological processes, **(D)** molecular functions, and **(E)** cellular components, together with **(F)** Kyoto Encyclopedia of Genes and Genomes (KEGG) pathway analysis. Dot size represents the number of genes associated with each term, and color indicates the adjusted statistical significance (FDR). **(G)** Volcano plot showing differential gene expression in HCE-2 cells following exposure to BEV-Se relative to control conditions. Differential expression was calculated using the same statistical approach.

Differential expression analysis identified 70 significantly upregulated and 28 downregulated transcripts following BEV-Pa treatment (FDR-adjusted p < 0.05; |log_2_FC| > 1) (**Figure 4A**; **Supplementary Table 3**). Interaction networks showed strong connectivity among inflammatory, immune and ECM-remodelling pathways (**Figure 4B**). Highly upregulated genes included *MMP9* (log_2_FC: 4.02) and *MMP12* (log_2_FC: 3.63), together with cytokines and receptors (*IL6*, *IL32*, *CCL22*, *CXCL1*, *CXCL8*), complement components (*CFB*, *C3*), and TNF receptors (*TNFRSF9*). Functional enrichment analysis highlighted pathways related to cytokine signaling, ligand–receptor interactions, and immune responses, including IL-17, TNF, and NF-κB signaling pathways, as well as pathways associated with infectious diseases such as legionellosis (**Figures 4C–F**). These gene products were predominantly associated with the secretome and extracellular compartments, including extracellular vesicles.

In contrast, BEV-Se induced a weaker and more restricted transcriptional response. Although 199 transcripts were expressed *de novo*, only 48 were protein-coding mRNAs, which did not show significant functional enrichment, whereas the remaining transcripts comprised pseudogenes, intronic transcripts, and long non-coding RNAs. Differential expression analysis (**Figure 4G**) identified only one significantly upregulated gene (*MMP9*) and six significantly downregulated transcripts (*KRT13*, PSG5, *LBH*, *MUC20P1*, *SLC2A14*, and *IGFL2-AS1*) (**Supplementary Table 3**).

These findings indicate that BEVs from pathogenic and commensal bacteria differentially shape epithelial behavior through distinct transcriptional programs.

### 3.3 Direct contact with *Pseudomonas aeruginosa* or *Staphylococcus epidermidis* elicits distinct transcriptomic responses in corneal epithelial cells

A co-culture model enabling direct interaction between corneal epithelial cells and two ocular-relevant bacterial species was established to assess transcriptional responses in epithelial cells under direct bacterial contact. A total of 12,434 transcripts were detected in control cells, 12,274 in cells exposed to *P. aeruginosa*, and 12,317 in those exposed to *S. epidermidis*, with 11,308 transcripts shared across all conditions (**Supplementary Figure 2A**). Transcriptional alterations were more pronounced following exposure to *P. aeruginosa*, as confirmed by principal component analysis (PCA) and heatmap clustering (**Supplementary Figures 2B, C**).

**Figure 5:**
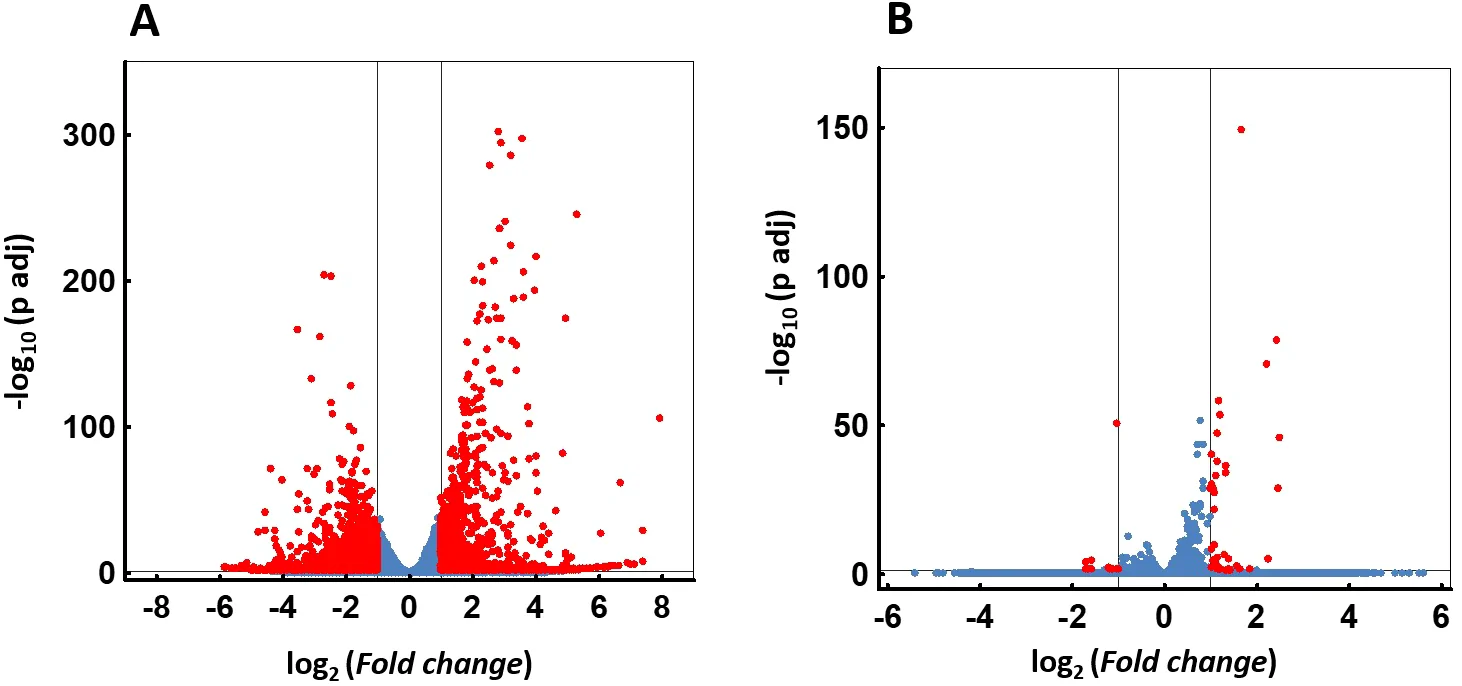
Differential gene expression in HCE-2 cells following direct bacterial contact. **(A, B)** Volcano plots showing differential gene expression in HCE-2 cells following direct contact with **(A)** *Pseudomonas aeruginosa* and **(B)** *Staphylococcus epidermidis* relative to control conditions. Each dot represents a gene; red dots indicate significantly regulated (adjusted *p* < 0.05 and |log_2_FC| > 1). Differential expression was calculated from n = 3 independent biological replicates using DESeq2 (negative binomial generalized linear model), and *p*-values were adjusted using the Benjamini–Hochberg false discovery rate (FDR) correction. **(C)** Distribution of RNA biotypes significantly altered in HCE-2 cells following exposure to bacteria or their extracellular vesicles, expressed as a percentage of the total. Coding RNAs are shown in orange and lncRNAs in blue.

Exposure to *P. aeruginosa* resulted in extensive transcriptional reprogramming, with 1,212 upregulated (75% protein-coding) and 1,670 downregulated transcripts (79% protein-coding) (**Figure 5A**). Notably, of the 256 genes previously induced by BEV-Pa (186 expressed *de novo* and 70 upregulated), only 54 remained upregulated upon direct bacterial contact, including key inflammatory mediators (*IL6*, *CXCL8*, *CXCL2*, *IL1A*), the receptor *TNFRSF11B*, and matrix metalloproteinases (*MMP1*, *MMP2*). In contrast, several BEV-induced genes associated with host defense, including *S100A8*, and *S100A9*, were downregulated under direct contact conditions.

In comparison, *S. epidermidis* induced a more limited transcriptional response, with 341 genes detected *de novo*. Consistent with vesicle-mediated responses, a lower proportion of altered transcripts corresponded to protein-coding genes (121 of 341), with a predominance of regulatory RNA species. Differential expression analysis identified only 37 significantly upregulated genes and 16 significantly downregulated transcripts (**Figure 5B**; **Supplementary Table 4**), consistent with a comparatively modest transcriptional response. Functional enrichment analysis of the upregulated genes indicated enrichment of immune- and inflammation-related pathways, including cytokine–receptor interactions and innate immune signaling pathways such as MAPK, IL-17, Toll-like receptor, NOD-like receptor, TNF, and PI3K–Akt signaling. However, no overlap was observed with the BEV-induced responses (**Supplementary Figure 3**).

Together, these results indicate that direct bacterial contact amplifies and reshapes epithelial transcriptional responses in a species-dependent manner. While *P. aeruginosa* induces a broad, protein-coding-dominated inflammatory program, *S. epidermidis* elicits a comparatively modest transcriptional response with limited differential expression despite the presence of numerous *de novo* regulatory RNA transcripts.

### 3.4 Corneal epithelial cells reshape exosomal cargo upon bacterial interaction

Exo-HCE cells were isolated under basal conditions and following co-culture with *P. aeruginosa* or *S. epidermidis*. Given the size overlap between exosomes and BEVs, isolation and separation procedures were optimized (Materials and Methods). The identity of the isolated vesicles and the absence of detectable BEV contamination were first evaluated by Western blot analysis. TSG101 was readily detected in all exosome preparations, whereas the bacterial BEV markers OprF (*P. aeruginosa*) and SepA (*S. epidermidis*) were detected only in the corresponding BEV preparations used as positive controls and were absent from Exo-CoPa and exosomes derived from *S. epidermidis*-exposed cells (Exo-CoSe) samples, supporting the absence of detectable bacterial vesicle contamination (**Supplementary Figure 4A**). To further validate the identity of the isolated vesicles, the LC–MS/MS dataset was compared with the recently proposed core exosomal and exclusion marker datasets described by Kugeratski et al. Twenty-six of the twenty-eight proposed core exosomal markers were identified, whereas only five of the twenty-one proposed exclusion markers were detected, including the absence of the classical negative marker calnexin (**Supplementary Table 1**). NTA showed that Exo-CoPa exhibited a main peak at ~167 nm and a representative particle concentration of approximately 8.55 × 10^9^ particles/mL, whereas Exo-CoSe displayed peaks at 91 and 127 nm with a representative particle concentration of approximately 2.33 × 10¹¹ particles/mL. These size distributions are consistent with those previously reported for epithelial-derived exosomes isolated from primary human limbal epithelial cultures using comparable isolation and characterization procedures (Verma et al., 2023). TEM confirmed the expected vesicular morphology (**Supplementary Figures 4B–E**).

Proteomic analysis identified 1,214 proteins shared across all conditions, with 379 proteins exclusively detected in control/Exo-CoSe samples and only eight unique to control/Exo-CoPa (**Supplementary Figure 5**). In Exo-CoPa, 162 proteins appeared *de novo* and 217 were lost (**Supplementary Table 5**). Functional enrichment analysis revealed a highly interconnected network associated with RNA splicing, mRNA processing, and RNA metabolism, suggesting incorporation of proteins involved in post-transcriptional regulation and cell cycle control (**Supplementary Figure 6A**). Conversely, proteins lost from Exo-CoPa were mainly associated with vesicular trafficking, extracellular interactions, and modules related to adhesion, migration, and ECM organization (**Supplementary Figure 6B**).

**Figure 6:**
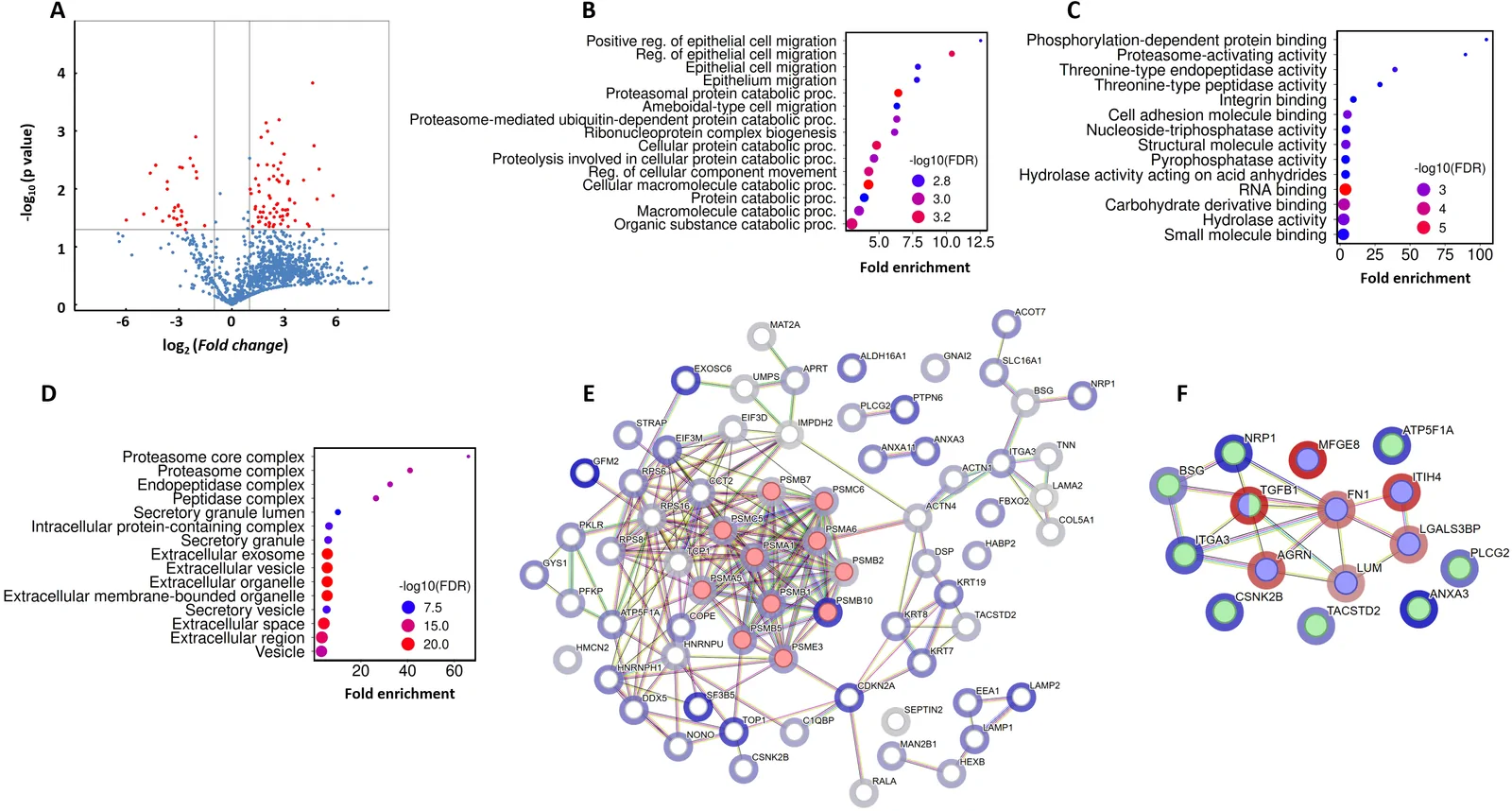
Differential proteomic profile and functional enrichment of Exo-CoPa. **(A)** Volcano plot showing differential protein abundance in exosomes derived from *Pseudomonas aeruginosa*-exposed HCE-2 cells (Exo-CoPa) relative to control conditions. Each dot represents a protein; red dots indicate significantly altered proteins (*p* < 0.05 and |log_2_FC| > 1). Differential protein abundance was determined from n = 3 independent biological replicates. Gene Ontology (GO) enrichment analysis of upregulated proteins showing **(B)** molecular functions, **(C)** cellular components, and **(D)** biological processes. Dot size represents the number of proteins associated with each term, and color indicates the adjusted statistical significance (FDR). **(E, F)** Protein–protein interaction networks of the altered proteins, predicted using the STRING database. The intensity of the halo reflects the degree of overexpression (blue) or underexpression (red) relative to control conditions. Distinct functional clusters are highlighted, including proteasome-associated proteins (red), epithelial migration-related proteins (green), and extracellular matrix components (purple). Proteasome-related proteins formed the most prominent interaction cluster.

Comparative proteomic analysis identified 100 proteins with altered abundance in Exo-CoPa (70 upregulated and 30 downregulated; *p* < 0.05; |log_2_FC| > 1) (**Figure 6A**). Upregulated proteins were strongly enriched in proteasome-mediated degradation pathways, including core catalytic and regulatory components (**Figures 6B–D**). KEGG analysis identified the proteasome pathway as the most significantly enriched (enrichment ratio: 68.1; FDR: 3.5 × 10^-14^), including multiple 20S core subunits (PSMB1, PSMB2, PSMB5, PSMB7, PSMA1, PSMA5, PSMA6), 19S regulators (Rpt6, Rpt4), and associated factors (PA28-γ, FBXO2, β2i) (**Figure 6E**). In parallel, proteins linked to epithelial adhesion and migration (BSG, ANXA3) were increased, whereas ECM components (LUM, AGR, FN) were reduced (**Figure 6F**).

**Figure 7:**
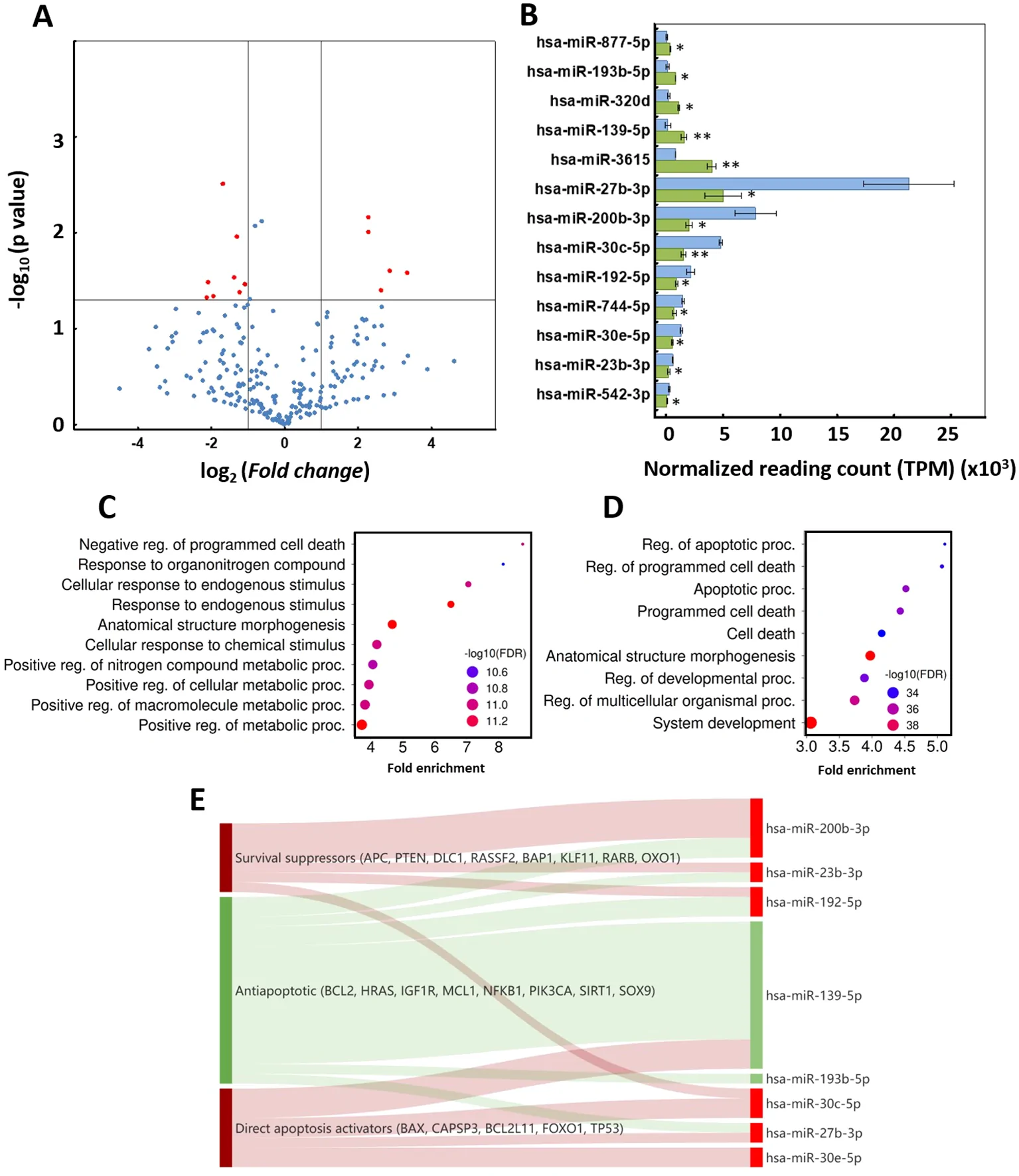
Differentially abundant miRNAs in Exo-CoPa and functional analysis of their experimentally validated target genes. **(A)** Volcano plot showing differential miRNA abundance in exosomes derived from *Pseudomonas aeruginosa*-exposed HCE-2 cells (Exo-CoPa) relative to control exosomes (Exo-HCE). Each dot represents a miRNA; red dots indicate significantly altered miRNAs (*p* < 0.05 and |log_2_FC| > 1). Differential miRNA abundance was determined from n = 3 independent biological replicates. **(B)** Transcript per million (TPM) values of miRNAs showing differential abundance between Exo-HCE (blue) and Exo-CoPa (green). Statistical significance is indicated as *p* < 0.05 (*) and *p* < 0.01 (**). Gene Ontology (GO) enrichment analysis of molecular functions associated with experimentally validated target genes of miRNAs whose abundance **(C)** increased or **(D)** decreased significantly. Dot size represents the number of target genes associated with each term, and color indicates the adjusted statistical significance (FDR). **(E)** Sankey diagram illustrating miRNA-mediated regulation of cell death–related pathways. On the left, target genes associated with anti-apoptotic functions are shown in green and pro-apoptotic genes in red. On the right, upregulated miRNAs are shown in green and downregulated miRNAs in red.

Analysis of RNA cargo in Exo-CoPa revealed extensive reorganization of non-coding RNA populations, characterized by marked increases in small nuclear RNAs (snRNAs; 31.5% to 45.6%) and small nucleolar RNAs (snoRNAs; 1.9% to 29.6%). Twenty-six miRNAs were lost, targeting 283 genes (**Supplementary Table 6**). Functional enrichment analysis of these targets revealed overrepresentation of DNA-binding functions and RNA polymerase II–dependent transcriptional regulation, with strong associations to nuclear structures such as chromatin, nucleoplasm and transcriptional complexes, as well as to cell adhesion and intercellular signaling components. Notably, significant enrichment was also observed in apoptosis and cell death pathways (**Supplementary Figure 7**). Among the miRNAs involved, hsa-miR-19b-3p was particularly relevant, as it targets key genes such as *PTEN* and *BCL2L11*, which are closely linked to the regulation of cell survival. Comparative analysis identified five up- and eight downregulated miRNAs (**Figures 7A, B**), targeting 56 and 238 genes, respectively (**Supplementary Table 7**). Functional analysis revealed a coordinated modulation of apoptosis-related pathways. Upregulated miRNAs were associated with negative regulation of cell death, whereas downregulated miRNAs targeted apoptosis-related genes, consistent with derepression of pro-apoptotic pathways (**Figures 7C–E**).

**Figure 8:**
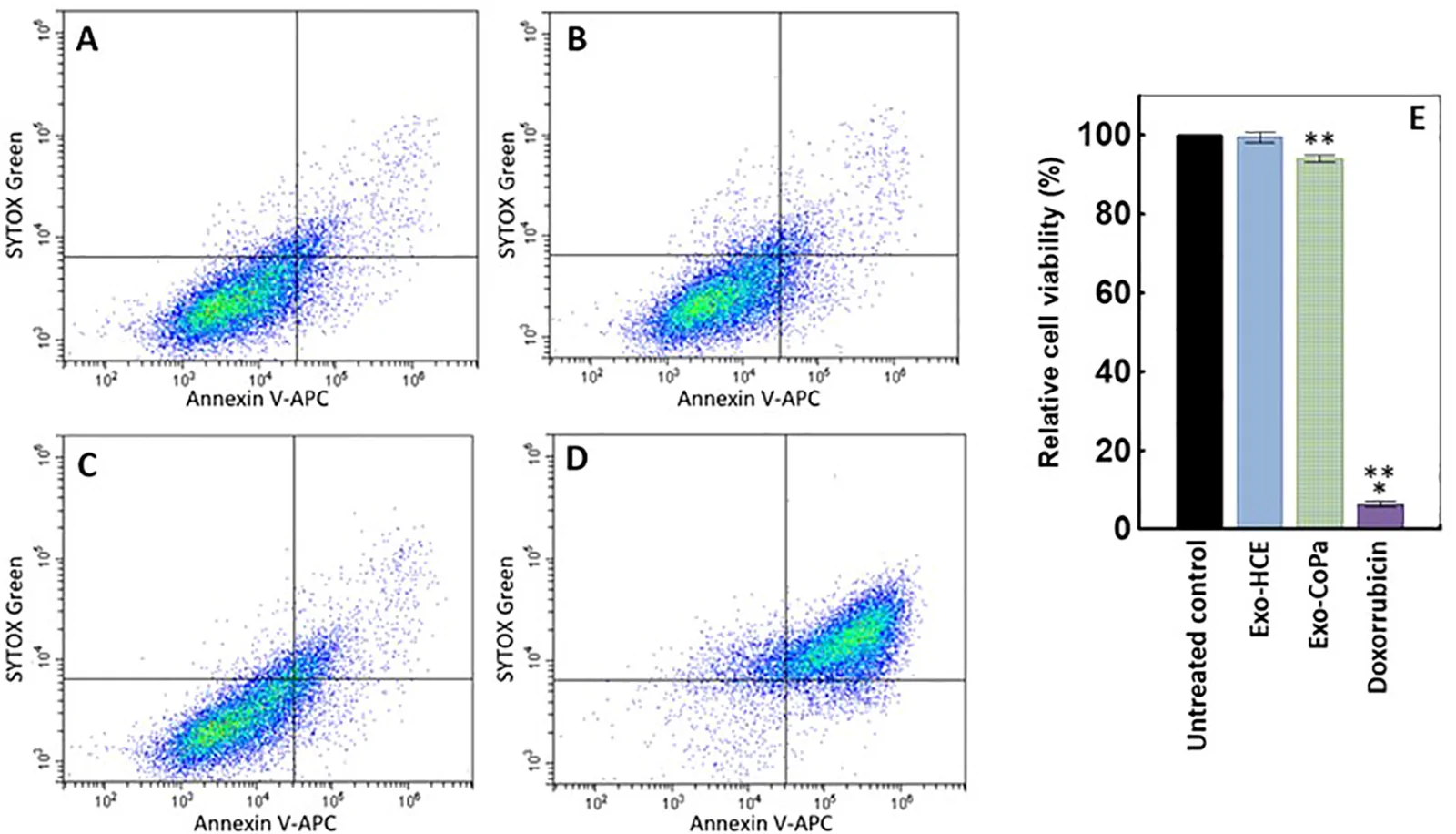
Validation of the pro-apoptotic activity of epithelial exosomes by flow cytometry. **(A–D)** Representative Annexin V/SYTOX Green dot plots of **(A)** untreated control cells and cells treated with **(B)** Exo-HCE, **(C)** Exo-CoPa, and **(D)** 5 μM doxorubicin (positive control). Cell populations were classified as viable (Annexin V−/SYTOX Green−), early apoptotic (Annexin V+/SYTOX Green−), late apoptotic (Annexin V+/SYTOX Green+), and necrotic (Annexin V−/SYTOX Green+). **(E)** Quantification of viable cells normalized to untreated controls. Exo-CoPa induced a significant reduction in cell viability compared with untreated cells, whereas Exo-HCE did not produce significant changes. Doxorubicin was used as a positive control. Data are presented as mean ± SD from n = 3 independent biological replicates. Statistical significance was assessed using the Mann–Whitney U test. p < 0.01 (**) and p < 0.001 (***).

To experimentally validate the bioinformatic prediction that Exo-CoPa may promote apoptotic responses, apoptosis was evaluated by Annexin V/SYTOX Green flow cytometry after exposure of HCE-2 cells to epithelial exosomes (**Figure 8**). Exo-HCE did not significantly affect cell viability compared with untreated controls (99.5%; p = 0.64), whereas Exo-CoPa induced a modest but significant reduction in cell viability (94.1%; p = 0.003). As expected, treatment with doxorubicin markedly reduced viability (6.1%; p < 0.001), confirming the sensitivity of the assay. These findings demonstrate that exosomes generated following interaction with *P. aeruginosa* acquire modest but significant pro-apoptotic activity, although the magnitude of the effect remained substantially lower than that induced by a classical apoptosis-inducing agent.

**Figure 9:**
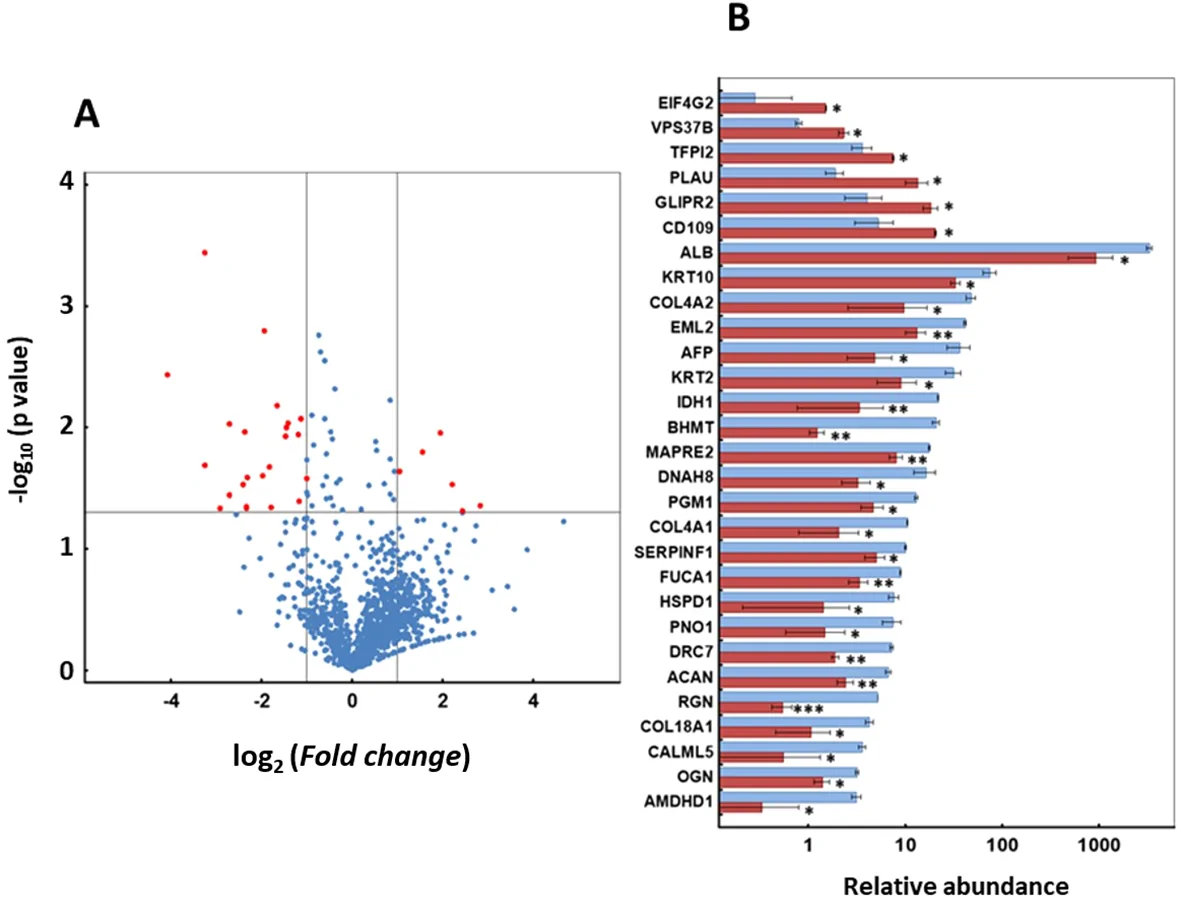
Differential proteomic profile of Exo-CoSe. **(A)** Volcano plot showing differential protein abundance in exosomes derived from *Staphylococcus epidermidis*-exposed HCE-2 cells (Exo-CoSe) relative to control conditions. Each dot represents a protein; red dots indicate significantly altered proteins (*p* < 0.05 and |log_2_FC| > 1). Differential protein abundance was determined from n = 3 independent biological replicates. **(B)** Relative abundance of proteins showing differential levels between Exo-HCE and Exo-CoSe. Statistical significance is indicated as *p* < 0.05 (*), p < 0.01 (**), and p < 0.001 (***).

In contrast, Exo-CoSe exhibited a more limited proteomic remodeling. A total of 55 proteins appeared *de novo*, including extracellular vesicle-related, adhesion-associated, and podosome-associated proteins, with enrichment of Rab33B, a GTPase involved in vesicle biogenesis and trafficking. Only four proteins were lost (RCN3, PCFH12, AOC1, NEDD4L) (**Supplementary Table 8**). Comparative analysis identified 29 proteins with altered abundance (6 upregulated and 23 downregulated; *p* < 0.05; |log_2_FC| > 1) (**Figure 9A**), including reduced levels of basement membrane collagens, aggrecan, and metabolic enzymes (**Figure 9B**).

RNA cargo analysis revealed a marked increase in miRNAs in Exo-CoSe (10% to 48.7%) (**Supplementary Table 9**). Fifty miRNAs appeared *de novo*, targeting 284 genes (**Supplementary Table 10**) involved in proliferation, differentiation, and cell cycle regulation, including targets such as *CCND1* and *CDK4/6/16*. Conversely, 21 miRNAs were lost, affecting 85 genes associated with immune signaling pathways, including *IL-6*, *TLR3/4*, and *JAK/STAT*. Cyclin inhibitors *CDKN1A* and *CDKN1B*, targets of hsa-miR-148a-5p and hsa-miR-149-3p, were also identified among these regulated genes.

**Figure 10:**
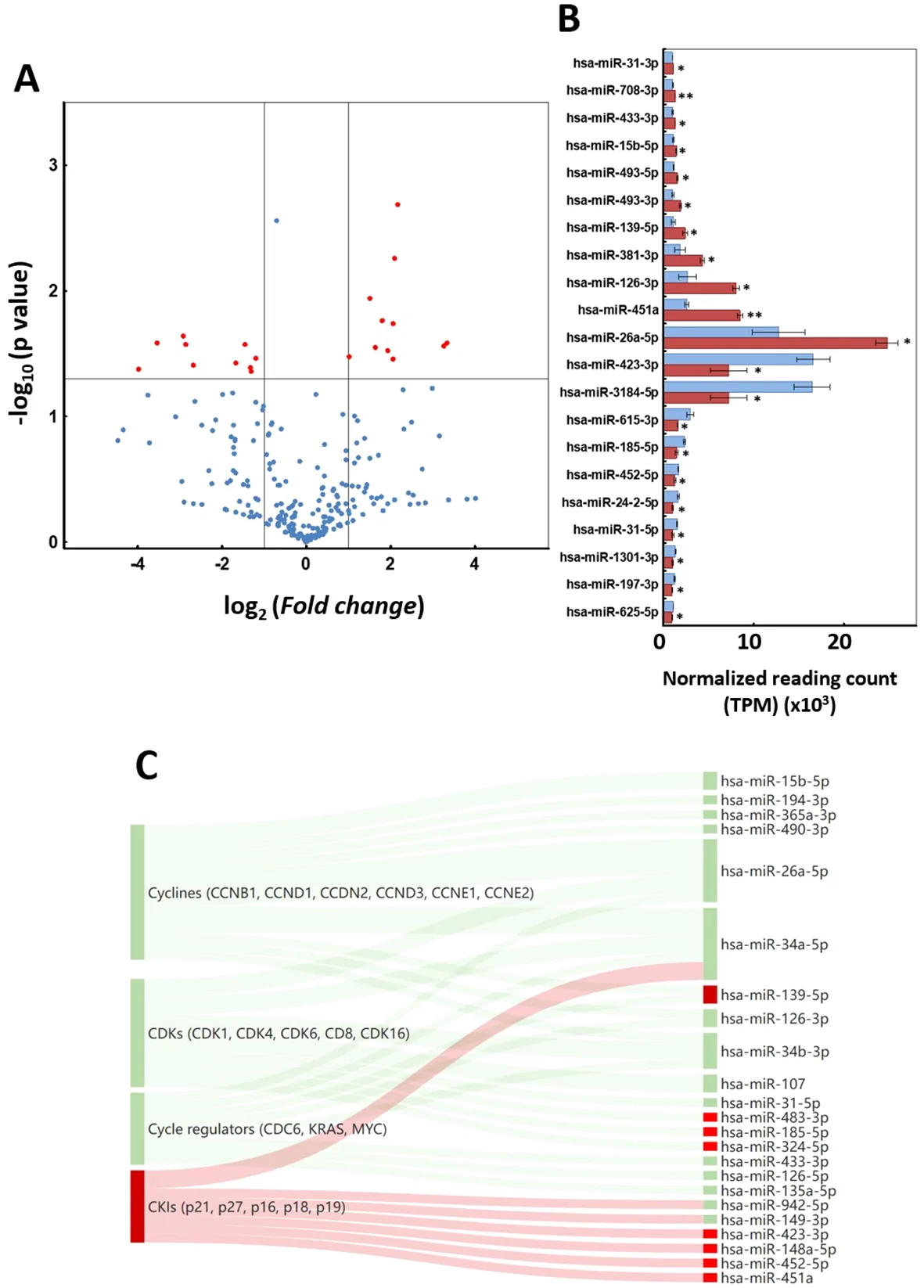
Differentially abundant miRNAs in Exo-CoSe and functional analysis of their experimentally validated target genes. **(A)** Volcano plot showing differential miRNA abundance in exosomes derived from *Staphylococcus epidermidis*-exposed HCE-2 cells (Exo-CoSe) relative to control exosomes (Exo-HCE). Each dot represents a miRNA; red dots indicate significantly altered miRNAs (*p* < 0.05 and |log_2_FC| > 1). Differential miRNA abundance was determined from n = 3 independent biological replicates. **(B)** Transcripts per million (TPM) values of miRNAs showing differential abundance between Exo-HCE (blue) and Exo-CoSe (red). Statistical significance is indicated as *p* < 0.05 (*) and *p* < 0.01 (**). **(C)** Sankey diagram illustrating miRNA-mediated regulation of cell cycle–related pathways. On the left, target genes associated with cell cycle activation are shown in green, and cell cycle inhibitors in red. On the right, upregulated miRNAs are shown in green and downregulated miRNAs in red.

Comparative analysis identified 21 altered miRNAs (11 upregulated and 10 downregulated) targeting 248 and 123 genes, respectively (**Figures 10A, B**; **Supplementary Table 10**). Genes targeted by upregulated miRNAs were enriched in cell proliferation-related pathways, including kinases such as *CCND1*, *CDK6* and *CDK8*, regulated by hsa-miR-26a-5p, as well as functions related to kinase activity, phosphorylation, signal transduction, plasma membrane organization and cell junction dynamics (**Figure 10C**). In contrast, genes targeted by downregulated miRNAs encoded proteins associated with epithelial development and differentiation, transcriptional and translational regulation, and membrane dynamics.

Functionally, these bacterially conditioned exosomes altered epithelial behavior. At 10^5^ exosomes per cell, both Exo-CoPa and Exo-CoSe reduced cellular metabolic activity, as determined by the MTT assay, with a stronger effect observed for Exo-CoSe (−16% at 48 h and −26% at 72 h relative to Exo-HCE), whereas Exo-CoPa induced more moderate reductions (−12% and −13% at 48 and 72 h, respectively) (**Supplementary Figures 8A, B**). Exo-CoPa also reduced wound closure (−25.6% at 6 h), whereas Exo-CoSe had no significant effect on cell motility (**Supplementary Figure 8C**).

To determine whether the epithelial responses observed in the co-culture model could be attributed to gentamicin treatment rather than bacterial interaction, HCE-2 metabolic activity was evaluated after exposure to gentamicin alone at the highest concentration used during the co-culture experiments (6.25 μg/mL). Gentamicin treatment alone did not significantly affect epithelial metabolic activity compared with untreated controls, whereas co-culture with *P. aeruginosa* induced a significant reduction (**Supplementary Figure 9**). These findings indicate that the reduced metabolic activity observed in the *P. aeruginosa* co-culture model is attributable to bacterial interaction rather than to gentamicin treatment alone.

## 4 Discussion

EV-mediated communication is increasingly recognized as a central mechanism in host–microorganism interactions, enabling the transfer of proteins and RNA that shape immune responses and tissue homeostasis (Toyofuku et al., 2019; Caruana and Walper, 2020; Kalluri and LeBleu, 2020; Margutti et al., 2024; Blanco-Agudín et al., 2025; Moghaddam et al., 2025). While this process has been extensively characterized in other systems, its role at the ocular surface remains poorly understood, despite the clinical relevance of microbial-driven inflammation in keratitis. In particular, how bacterial extracellular vesicles (BEVs) and epithelial-derived exosomes integrate to coordinate epithelial responses during early host–microorganism interactions remains unclear.

In this study, BEVs derived from two clinically relevant ocular microorganisms, *P. aeruginosa* and *S. epidermidis*, together with epithelial exosomes, were isolated and characterized. The inclusion of both *P. aeruginosa* and *S. epidermidis* was intended to compare two biologically relevant but ecologically distinct ocular-associated bacteria, representing pathogenic and commensal interactions, respectively, rather than microorganisms with equivalent virulence. Our results demonstrate that BEVs act as active signaling mediators capable of modulating corneal epithelial cell behavior in the absence of live bacteria, and that these effects are strongly species-dependent.

NTA and TEM confirmed that vesicle size and mean diameter were within the expected range (Kalluri and LeBleu, 2020; Margutti et al., 2024). Some samples showed bimodal size distributions, likely reflecting residual larger vesicles that are overrepresented in NTA because of greater light scattering (Samaeekia et al., 2018; Lozano et al., 2022). However, TEM confirmed the typical homogeneous morphology of all vesicle preparations.

One potential limitation of studies involving epithelial cells exposed to bacteria is the possible carry-over of BEVs during exosome isolation, given the overlapping size distribution of both vesicle populations. To address this issue, we combined complementary characterization approaches. Western blot analysis confirmed the presence of the exosomal marker TSG101, whereas the bacterial BEV markers OprF (*P. aeruginosa*) and SepA (*S. epidermidis*) were detected only in the corresponding BEV preparations used as positive controls and were absent from Exo-CoPa and Exo-CoSe samples. In addition, LC–MS/MS analysis identified 26 of the 28 proposed core exosomal markers recently described by Kugeratski et al., while only five of the 21 proposed exclusion markers were detected, including the absence of the classical negative marker calnexin (CANX). Although the LC–MS/MS search was performed exclusively against the human proteome, these complementary findings, together with NTA and TEM characterization, support the conclusion that the analyzed proteome predominantly represents host-derived epithelial exosomes and indicate minimal, if any, bacterial extracellular vesicle carry-over.

BEV-Pa induced a robust inflammatory transcriptional program characterized by cytokine production and innate immune signaling. This response included *IL6*, *IL32*, and several CXCL chemokines, particularly *CXCL10* and *CXCL11*, which are associated with neutrophil chemotaxis and CXCR3-dependent T-cell recruitment (Ayilam Ramachandran et al., 2023; Gao et al., 2018), consistent with previous reports describing pro-inflammatory responses induced by *P. aeruginosa*-derived extracellular vesicles in epithelial cells (Ayilam Ramachandran et al., 2023). It also increased the expression of *S100A8* and *S100A9*, damage-associated molecular patterns (DAMPs) with a recognized protective role in ocular defense against *P. aeruginosa*, whose inhibition is associated with more severe keratitis (Gao et al., 2013; Chidambaram et al., 2017). In parallel, *MMP1*, *MMP2*, *MMP9* and *MMP12* were markedly upregulated, indicating extracellular matrix degradation and remodeling; notably, *MMP9* showed more than a 16-fold increase, consistent with its strong induction in bacterial and fungal keratitis (Lapp et al., 2024). These molecular changes were accompanied by increased epithelial motility, suggesting that ECM remodeling contributes to epithelial repair. Together, these findings indicate that BEV-Pa not only triggers inflammatory responses but also promotes early tissue remodeling before direct bacterial contact, supporting its role as an early signaling mediator.

By contrast, BEV-Se induced a substantially milder transcriptional response characterized by limited differential expression after FDR correction and a predominance of newly detected regulatory RNA species rather than widespread changes in protein-coding genes. Although only a single protein-coding gene remained significantly upregulated after multiple-testing correction, the predominance of newly detected non-coding and regulatory RNA species suggests that BEV-Se primarily influences post-transcriptional regulatory processes rather than inducing a broad inflammatory transcriptional program. These findings are consistent with the lower virulence and predominantly commensal behavior generally associated with *S. epidermidis*. However, our results suggest that the observed differences are not solely quantitative. Rather than simply representing an attenuated version of the response induced by *P. aeruginosa*, BEV-Se was associated with a distinct transcriptional profile characterized predominantly by the appearance of regulatory RNA species and only minimal changes in protein-coding gene expression after multiple-testing correction. Together with our recent observations demonstrating species-specific regulation of corneal epithelial glycosaminoglycan biosynthesis by different ocular-associated bacteria and their extracellular vesicles (Blanco-Agudín et al., 2026a, b), these findings support the concept that epithelial cells discriminate between microbial species through qualitatively distinct vesicle-mediated communication mechanisms rather than responding exclusively according to bacterial virulence.

Direct bacterial contact amplified these species-specific differences. Exposure to *P. aeruginosa* induced extensive transcriptional reprogramming involving nearly 2,900 differentially expressed transcripts, with persistent activation of inflammatory mediators and ECM-remodeling genes previously induced by BEV-Pa. Interestingly, the reduction of *S100* family genes after direct contact suggests a two-phase response: an initial BEV-mediated activation of epithelial defense followed by bacterial modulation of host antimicrobial mechanisms to facilitate persistence (Gao et al., 2013; Chidambaram et al., 2017). This pattern highlights the temporal complexity of host–pathogen interactions and suggests that BEVs contribute to the early phase of epithelial activation, while direct bacterial contact introduces additional regulatory layers.

A major finding of this study is the extensive remodeling of exosomal cargo following bacterial exposure. Although only TSG101 was used as the positive marker for routine Western blot validation, the subsequent LC-MS/MS proteomic characterization demonstrated the presence of 26 of the 28 recently proposed core exosomal markers together with the absence of the classical exclusion marker calnexin, providing strong additional support for the exosomal enrichment of our preparations. Exo-CoPa showed marked enrichment of proteins involved in RNA processing and proteasome-mediated degradation. The accumulation of spliceosomal components and RNA metabolism-associated proteins, together with the redistribution of snRNAs and snoRNAs, indicates activation of post-transcriptional regulatory pathways, a pattern previously associated with infectious and stress responses (Karna et al., 2016; Slonchak et al., 2019; Huang et al., 2025; Rozek et al., 2025). These changes suggest that epithelial cells actively reprogram exosomal cargo, potentially contributing to the propagation of stress-related signals within the epithelium.

Particularly notable was the enrichment of proteasome components, including catalytic core subunits, regulatory ATPases and the β2i immunoproteasome subunit. This finding is highly relevant, as the proteasome is a central regulator of stress responses, inflammation, epithelial homeostasis and apoptosis (Bard et al., 2018; Mohapatra et al., 2021). Similar proteasome activation signatures have been described in intestinal and pulmonary bacterial infection models (Kishta et al., 2017; Mohapatra et al., 2021), and we previously reported related findings in bronchial epithelial exosomes exposed to *P. aeruginosa* (Lozano-Iturbe et al., 2024). The presence of β2i further suggests active epithelial participation in antigen processing and inflammatory signaling (Kimura et al., 2015).

Consistent with these findings, Exo-CoPa also showed miRNA changes compatible with derepression of pro-apoptotic pathways. The loss or downregulation of miRNAs such as hsa-miR-19b-3p and miR-30c-5p, together with the increase of miRNAs targeting anti-apoptotic regulators, is consistent with the experimentally validated pro-apoptotic activity of Exo-CoPa and supports activation of epithelial apoptosis as part of the host response to bacterial challenge (Häcker, 2018; Guindolet and Gabison, 2020; Abbas and Larisch, 2021; Jiang et al., 2021; Li et al., 2021).

The enrichment of proteasome-related proteins in Exo-CoPa would suggest that exosomes may function as vehicles for the dissemination of proteostasis-related signals during infection, contributing to coordinated epithelial responses and potentially amplifying inflammatory signaling in neighboring cells. The predicted enrichment of apoptosis-related pathways based on Exo-CoPa miRNA cargo was further supported by functional validation. Annexin V/SYTOX Green analysis demonstrated a modest but significant reduction in epithelial cell viability following exposure to Exo-CoPa, whereas basal epithelial exosomes did not induce apoptosis. These findings indicate that the altered molecular cargo of Exo-CoPa is accompanied by measurable biological activity and reinforce the conclusion that epithelial exosomes released after interaction with *P. aeruginosa* contribute to the propagation of pro-apoptotic signaling. Although the apoptotic response was considerably weaker than that induced by doxorubicin, these results support our bioinformatic analyses and indicate that Exo-CoPa participate in the amplification, rather than the initiation, of epithelial damage.

This coordinated modulation of miRNA networks suggests that epithelial exosomes actively contribute to the propagation of stress-related signaling beyond the initially exposed cells. Exo-CoPa also induced functional alterations in recipient epithelial cells, reducing cellular metabolic activity and impairing wound closure, indicating that exosome-mediated communication contributes to the dissemination of stress and inflammatory signals across the epithelium. These findings are consistent with the emerging role of corneal miRNAs in regulating epithelial homeostasis, inflammation, wound healing, and cell survival, and further support the concept that exosomal miRNAs may act as mechanistic mediators and candidate biomarkers of ocular surface disease (Verma et al., 2025c).

In contrast, direct exposure to *S. epidermidis* induced more limited transcriptional and exosomal remodeling, consistent with its commensal nature and lower virulence. The limited overlap between responses to the bacterium and to its BEVs indicates that corneal epithelial cells respond differently depending on whether they encounter BEVs or the live bacterium. Enrichment of immune-related pathways, including IL-17, MAPK, Toll-like receptor and NOD-like receptor signaling, suggests epithelial recognition of *S. epidermidis* despite the comparatively modest transcriptional response. As with BEV-Se, the predominance of altered ncRNAs and regulatory transcripts supports post-transcriptional and epigenetic regulation as a major feature of the response to *S. epidermidis*. This suggests that, under commensal conditions, epithelial responses are primarily regulated through post-transcriptional mechanisms that fine-tune cell behavior rather than triggering strong inflammatory activation.

At the exosomal level, *S. epidermidis* induced moderate remodeling of both protein and ncRNA cargo. Proteins involved in adhesion and membrane interactions were enriched, including CD109 and other molecules potentially involved in host–bacteria interactions (Schmidt et al., 2015; Zhang et al., 2019; Caballero et al., 2021; Rühling et al., 2025). More importantly, the strong increase in exosomal miRNAs targeting cell-cycle regulators, including *CCND1* and *CDK* family members (Bertero et al., 2011; Cheng et al., 2021; Wu et al., 2022; Ueno et al., 2023), was consistent with the observed reduction in epithelial cellular metabolic activity. This functional association supports the biological relevance of exosome-mediated ncRNA transfer and suggests that altered exosomal cargo contributes to the modulation of epithelial homeostasis. These findings are consistent with the emerging role of corneal miRNAs as regulators of epithelial homeostasis and tissue repair and further support their potential utility as candidate biomarkers of ocular surface disease (Verma et al., 2025c).

Interestingly, BEV-Se initially promoted a more regulatory and partially anti-inflammatory response. However, direct bacterial contact induced exosomal remodeling compatible with derepression of pro-inflammatory pathways, including TLR3- and JAK/STAT-associated signaling (Guo et al., 2020). This observation highlights the dynamic and context-dependent nature of epithelial responses to commensal microorganisms. Notably, the comparatively restrained response induced by *S. epidermidis* suggests that not all bacterial EV-mediated interactions are intrinsically pro-inflammatory, but may instead contribute to epithelial adaptation to low-virulence microbial exposure.

Taken together, these findings support a model in which EVs act as central coordinators of epithelial responses to microbial stimuli. BEVs provide an early signal that activates defense and remodeling pathways, whereas exosomes produced by epithelial cells propagate and modulate these signals within the tissue. The species-specific nature of these responses further indicates that EV-mediated communication contributes to the discrimination between pathogenic and commensal microorganisms, thereby playing a key role in maintaining ocular surface homeostasis. Although bacterial virulence undoubtedly contributes to the magnitude of epithelial responses, the distinct transcriptomic and exosomal remodeling patterns observed for each bacterial species indicate that EV-mediated communication cannot be explained solely by differences in pathogenic potential. Rather, these findings support the existence of species-specific signaling programs that shape host epithelial responses according to microbial identity. Importantly, this study was conceived as a systems-level analysis of EV-mediated communication rather than as a reductionist dissection of individual pathways. In this context, the convergence of transcriptomic, proteomic, functional and ncRNA datasets strongly supports the existence of coordinated biological reprogramming in corneal epithelial cells exposed to microbial and vesicle-derived stimuli.

This study has several limitations. The analyses were performed using an immortalized corneal epithelial cell line and *in vitro* interaction models, which cannot fully reproduce the complexity of the ocular surface environment. In addition, only two ocular-associated bacterial species were analyzed. Furthermore, the *P. aeruginosa* co-culture model required a higher gentamicin concentration and recurrent medium replacement to control bacterial growth while maintaining a stable bacterial load and preserving epithelial cell viability. Although gentamicin alone did not significantly affect epithelial metabolic activity under the conditions tested, potential effects on exosome release or molecular cargo cannot be completely excluded. Likewise, although the isolated vesicles were extensively characterized by Western blot, NTA, TEM, and LC–MS/MS, and no bacterial BEV contamination was detectable using the analytical approaches employed, complete exclusion of BEV carry-over cannot be unequivocally demonstrated because of the overlapping physicochemical properties of host- and bacteria-derived vesicles. Moreover, the clinical translation of EV-based diagnostics and therapeutics will require harmonized quality control, potency assessment, manufacturing (CMC), and regulatory standards, together with rigorous characterization and standardized analytical workflows to ensure product quality, reproducibility, and regulatory compliance (Verma and Arora, 2025a). These challenges reflect broader methodological considerations associated with EV isolation and characterization that remain active areas of investigation in the field (Verma et al., 2025b). Continued efforts toward standardized quality control, EV characterization, reproducible isolation workflows, and transparent data reporting will be essential to improve reproducibility and facilitate future clinical implementation (Verma et al., 2025c).

## 5 Conclusions

Our findings demonstrate that interactions between the corneal epithelium and ocular-associated bacteria reprogram vesicle-mediated communication in a species-specific manner. *P. aeruginosa* induces a robust inflammatory and proteasome-related pathways, whereas *S. epidermidis* elicits a substantially more limited transcriptional response characterized by the predominance of regulatory non-coding RNA species and distinct exosomal remodeling. Our findings further demonstrate that BEVs act as early signaling mediators, while epithelial-derived exosomes contribute to the propagation and modulation of these responses across the tissue by acquiring distinct molecular cargo and biological activities, including impaired epithelial wound closure and a modest but significant pro-apoptotic activity following *P. aeruginosa* exposure. These findings identify EVs as key intermediates of host–microorganism communication at the ocular surface, linking early bacterial signaling to coordinated epithelial reprogramming, and highlight their potential relevance as biomarkers and therapeutic targets in ocular surface disease.

## Data Availability

The datasets presented in this study can be found in online repositories. The names of the repository/repositories and accession number(s) can be found below: https://www.ncbi.nlm.nih.gov/geo/, GSE334089 https://www.ncbi.nlm.nih.gov/geo/, GSE334176.
