# Supplementary material for: Bacterial extracellular vesicles and direct microbial contact drive species-specific corneal epithelial reprogramming
Source: Front Cell Infect Microbiol. 2026 Aug 12;16:1899147. doi: 10.3389/fcimb.2026.1899147 (PMC13506776; doi:10.3389/fcimb.2026.1899147)
Supplement: Supplementary file 1 [file SupplementaryFile1.pdf]

Bacterial extracellular vesicles and direct microbial contact drive species-specific corneal epithelial reprogramming

Noelia Blanco-Agudín, Suhui Ye, Sara González-Fernández, Iván Fernández-Vega, Jesús Merayo-Lloves, Luis M. Quirós

Supplementary Figures

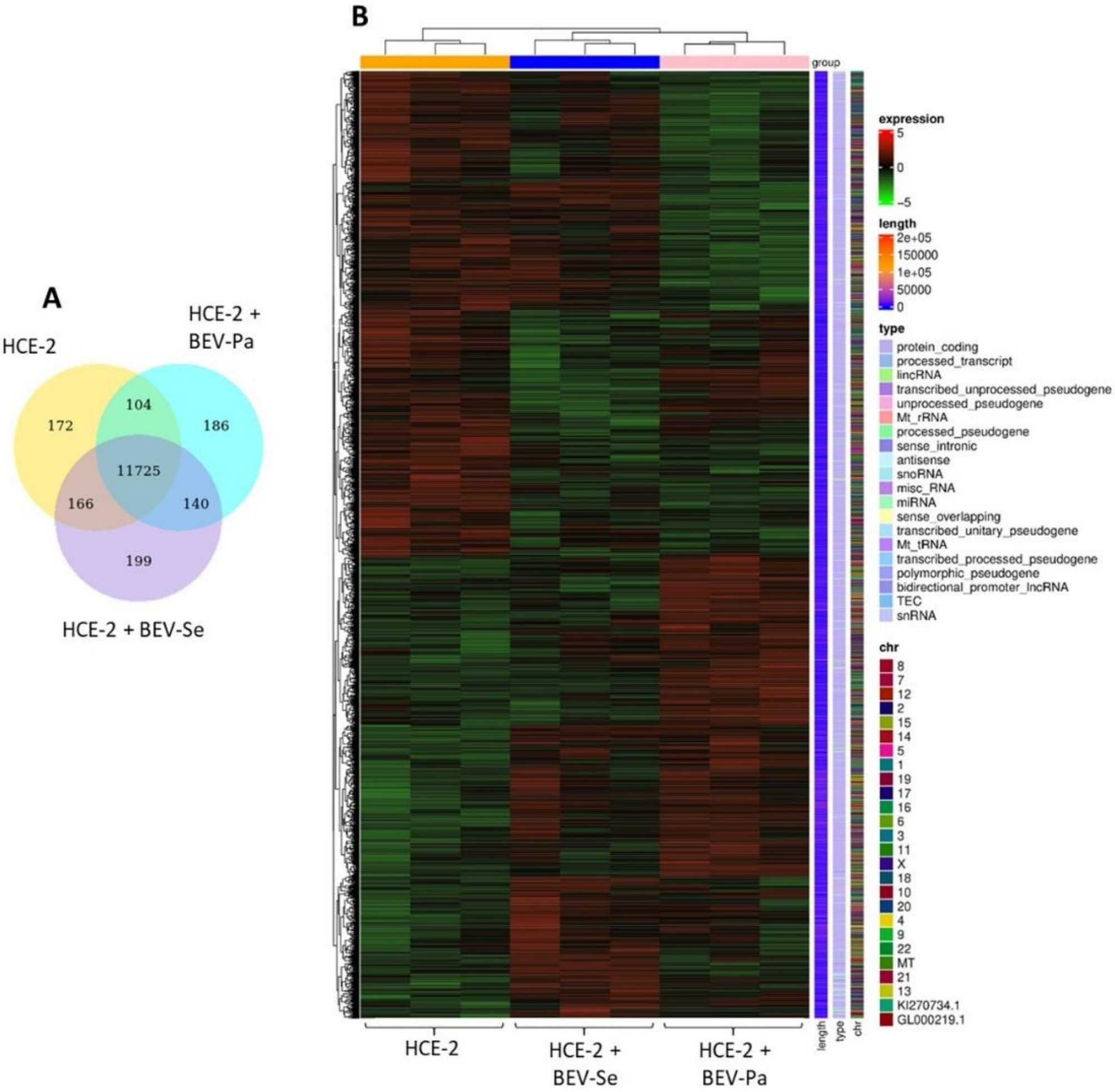

Supplementary Figure 1. Species-specific gene expression patterns induced by BEVs in HCE-2 cells. **(A)** Venn diagram showing the number of differentially expressed genes as well as those shared between conditions. **(B)** Heatmap displaying hierarchical clustering of samples based on gene expression profiles induced by BEVs from each microorganism.

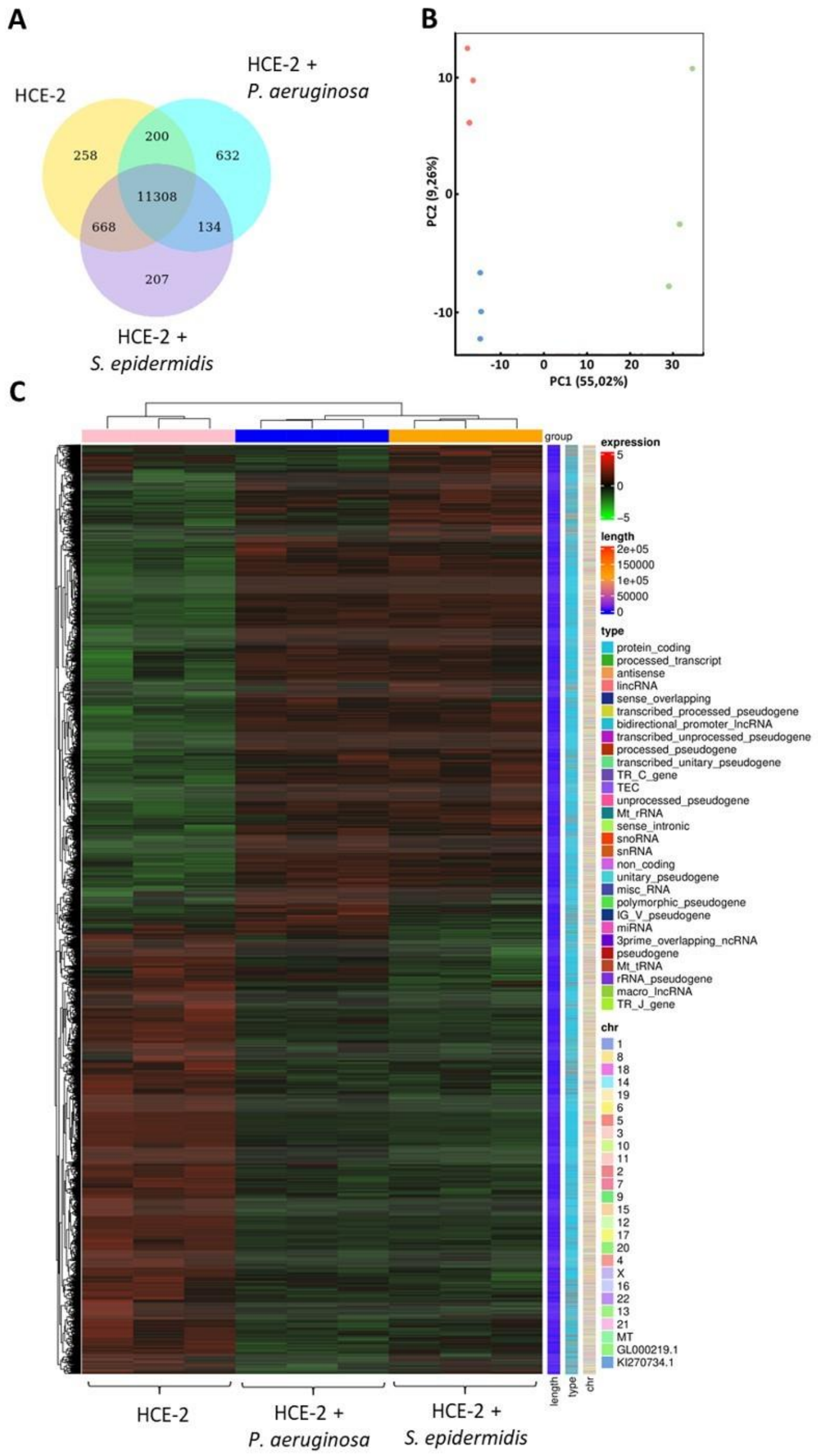

Supplementary Figure 2. Gene expression analysis of HCE-2 cells following exposure to *P. aeruginosa* and *S. epidermidis*. **(A)** Venn diagram showing the number of differentially expressed genes as well as those shared between conditions. **(B)** Principal component analysis (PCA) of gene expression across the three conditions: HCE-2 (red), HCE-2 + *P. aeruginosa* (green) and HCE-2 + *S. epidermidis* (blue). **(C)** Heatmap displaying hierarchical clustering of samples based on gene expression profiles induced by each microorganism.

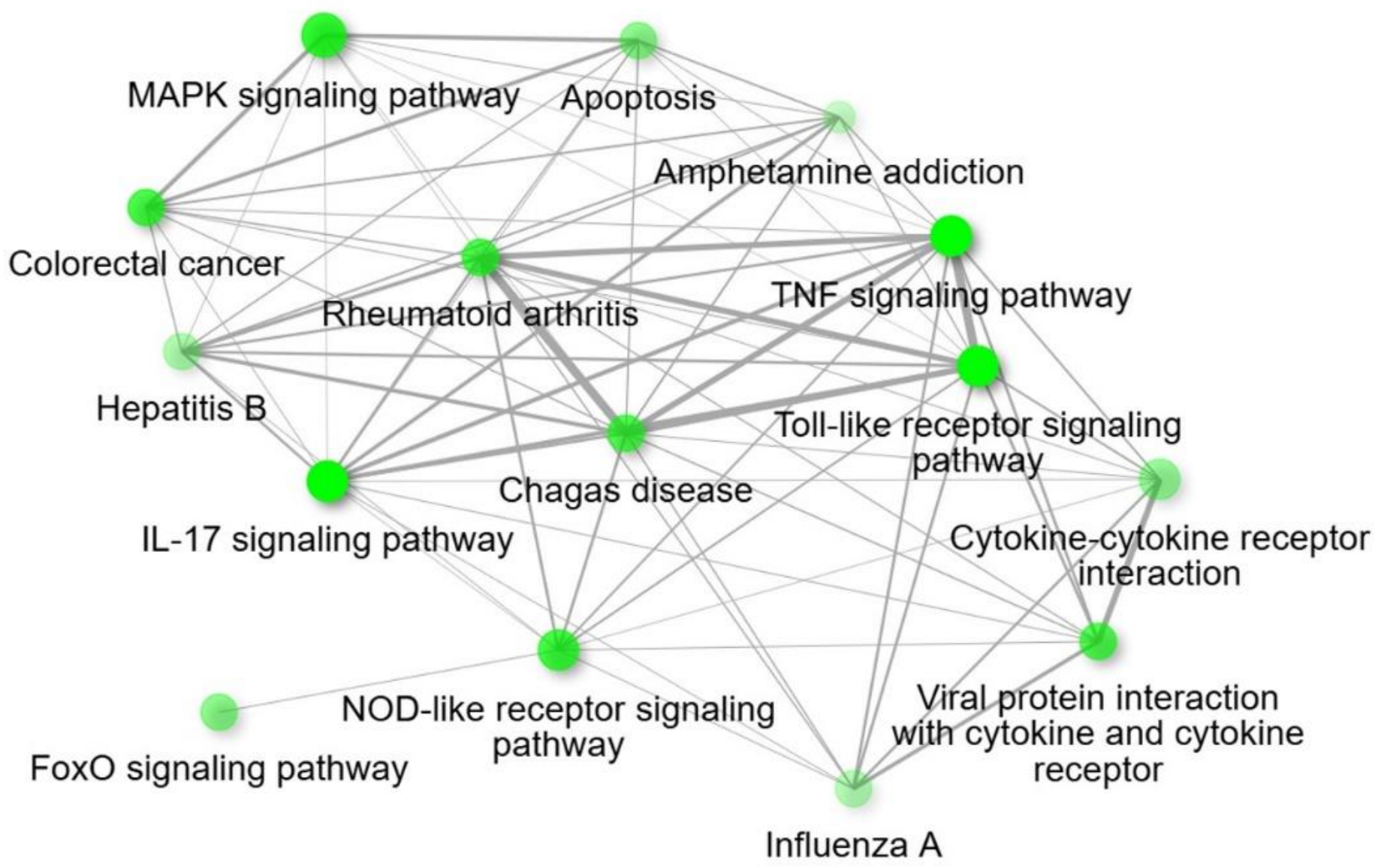

Supplementary Figure 3. KEGG enrichment network of genes upregulated in HCE-2 cells following exposure to *S. epidermidis*. Each node corresponds to a biological process, and connections indicate at least 20% gene overlap between terms. Node size is proportional to the number of associated proteins, and color intensity reflects the statistical significance of enrichment ($-\log_{10}$ FDR). Edge thickness indicates the number of genes shared between terms.

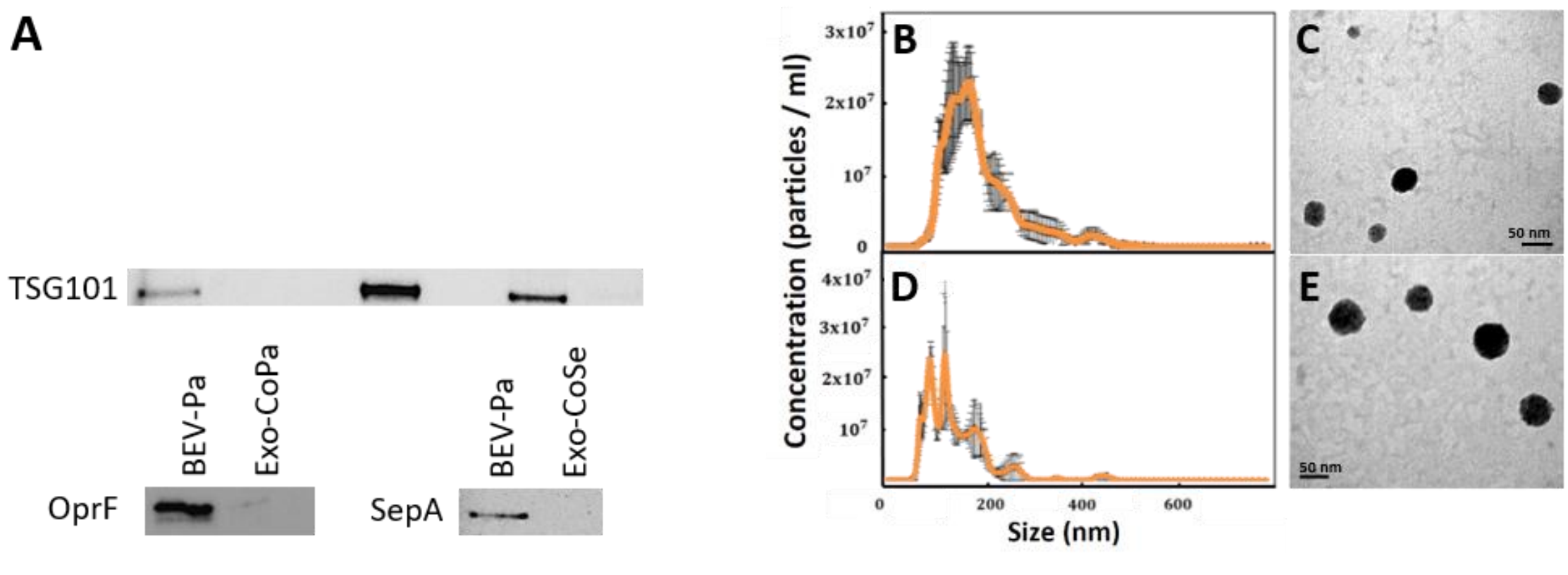

Supplementary Figure 4. Purification and characterization of exosomes from HCE-2 cells co-cultured with bacteria. **(A)** Western blot analysis using TSG101 as exosome marker, OprF as BEV-Pa marker and SepA as BEV-Se marker. NTA size distribution of **(B)** Exo-CoPa and **(D)** Exo-CoSe, and TEM images of **(C)** Exo-CoPa and **(E)** Exo-CoSe.

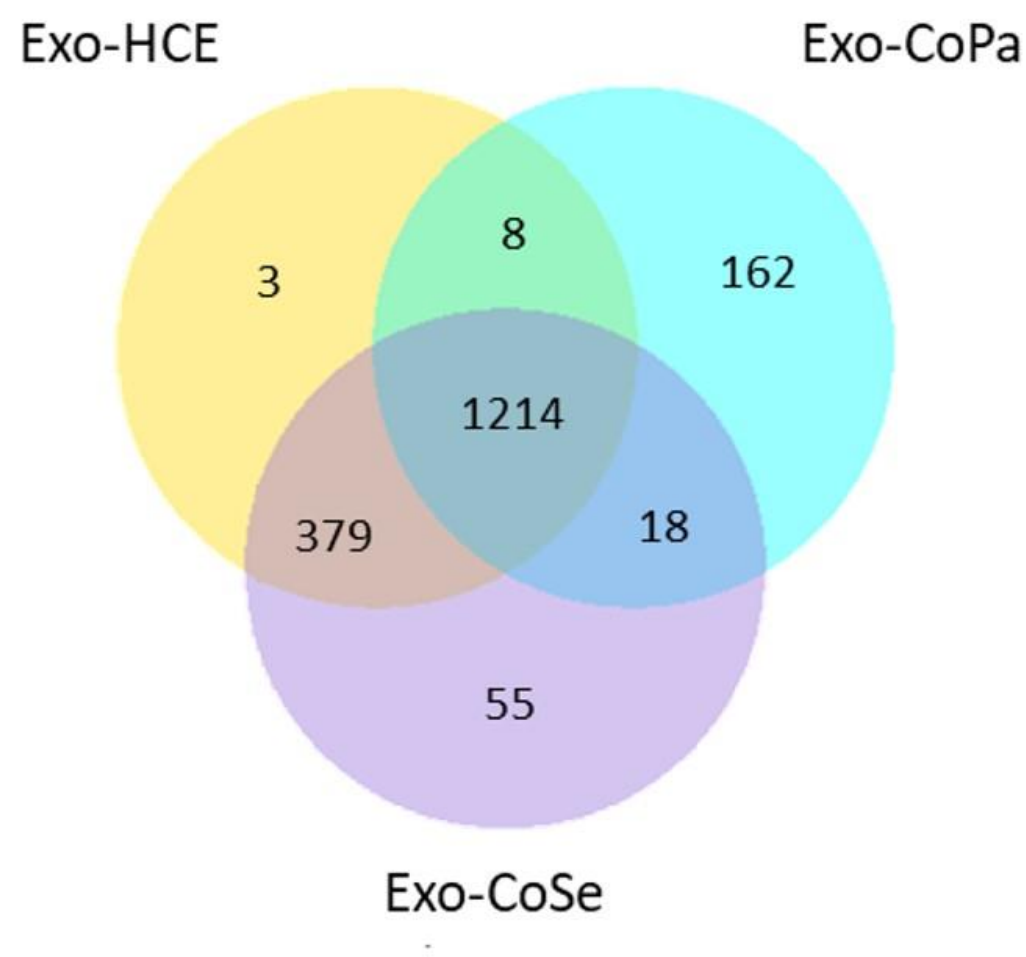

Supplementary Figure 5. Venn diagram showing the number of differentially expressed genes and those shared among Exo-HCE, Exo-CoPa and Exo-CoSe.

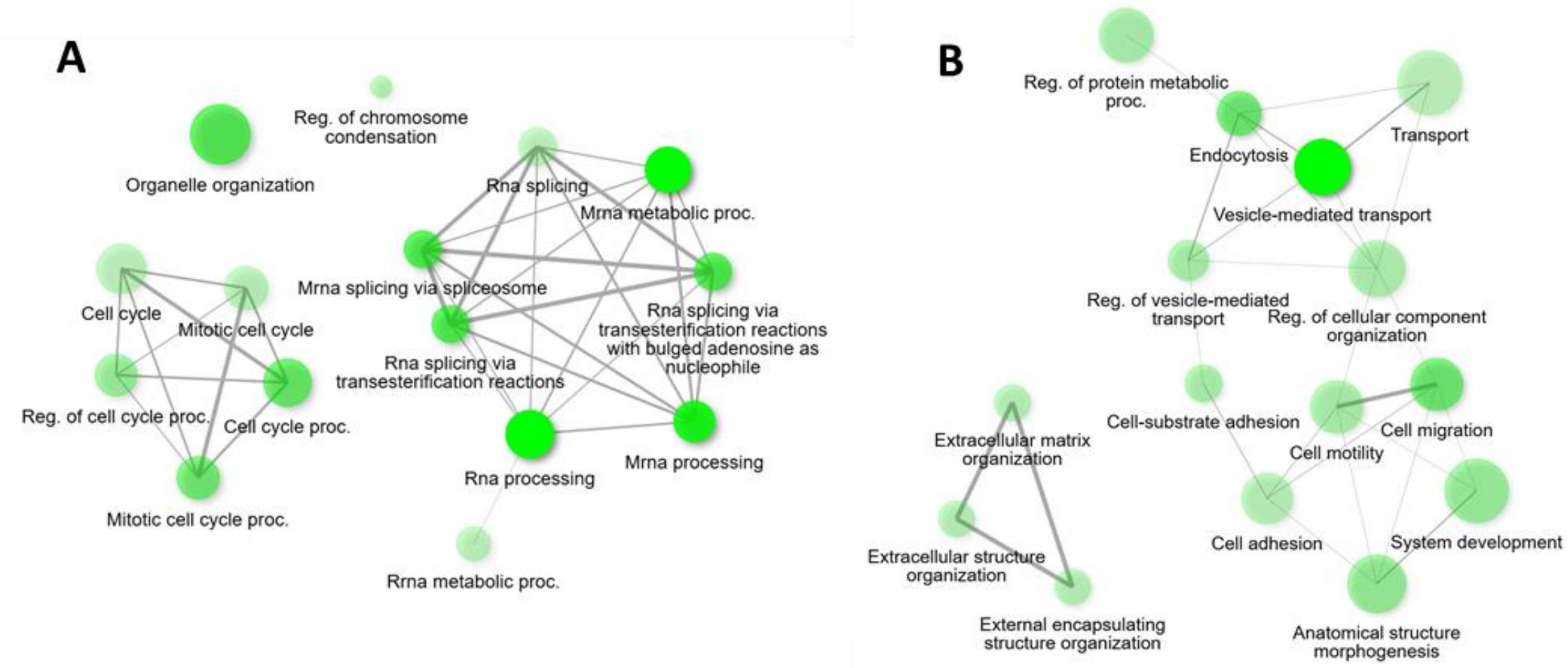

Supplementary Figure 6. GO functional enrichment network of proteins detected **(A)** *de novo* and **(B)** lost in Exo-CoPa. Each node corresponds to a biological process, and connections indicate at least 20% gene overlap between terms. Node size is proportional to the number of associated proteins, and color intensity reflects the statistical significance of enrichment ($-\log_{10}$ FDR). Edge thickness indicates the number of genes shared between terms.

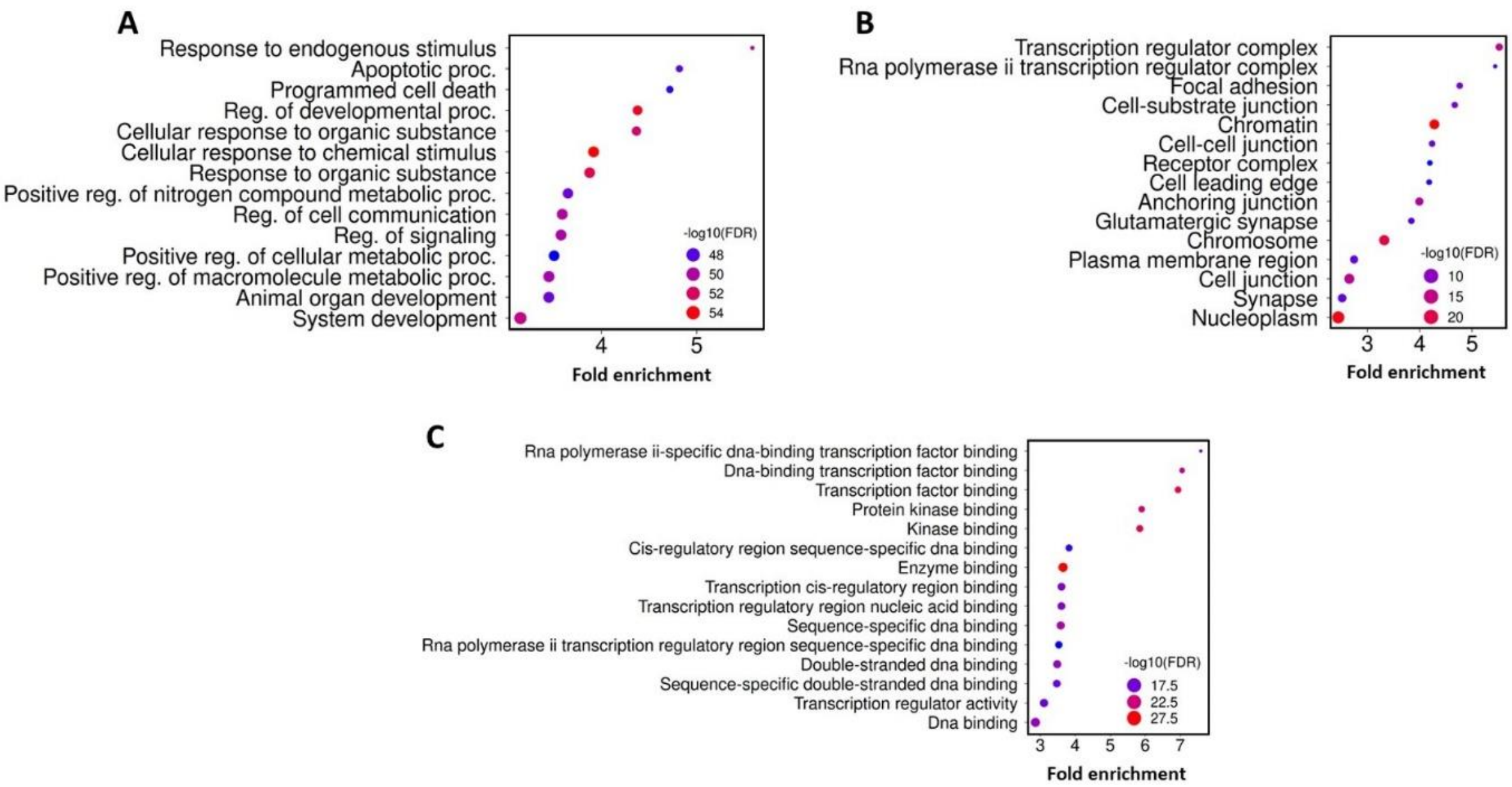

Supplementary Figure 7. GO functional analysis of target genes of miRNAs undetected in Exo-CoPa compared with Exo-HCE, including the categories **(A)** biological process, **(B)** cellular component and **(C)** molecular function.

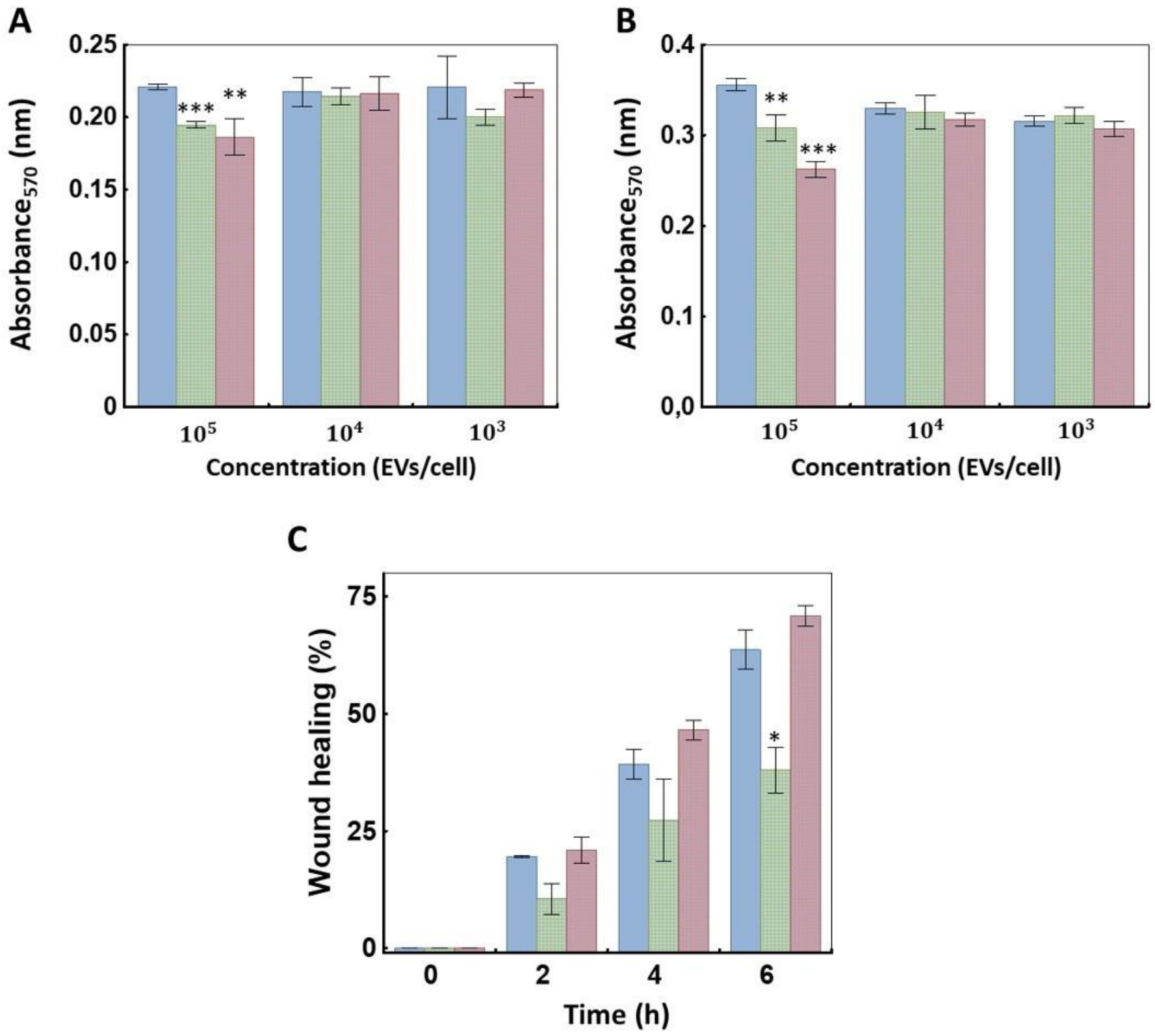

Supplementary Figure 8. Functional effects of epithelial exosomes on HCE-2 cell metabolic activity and wound healing. Cellular metabolic activity of HCE-2 cells following treatment with Exo-HCE (blue), Exo-CoPa (green), or Exo-CoSe (red) at different exosome-to-cell ratios after **(A)** 48 h and **(B)** 72 h, assessed by MTT assay. Data are presented as mean ± SD from n = 5 independent biological replicates. **(C)** Quantification of wound closure in HCE-2 cells treated with epithelial exosomes. Data are presented as mean ± SD from n = 3 independent biological replicates. Statistical significance was assessed using the Mann–Whitney U test. $p < 0.05$ (*), $p < 0.01$ (**) and $p < 0.001$ (***).

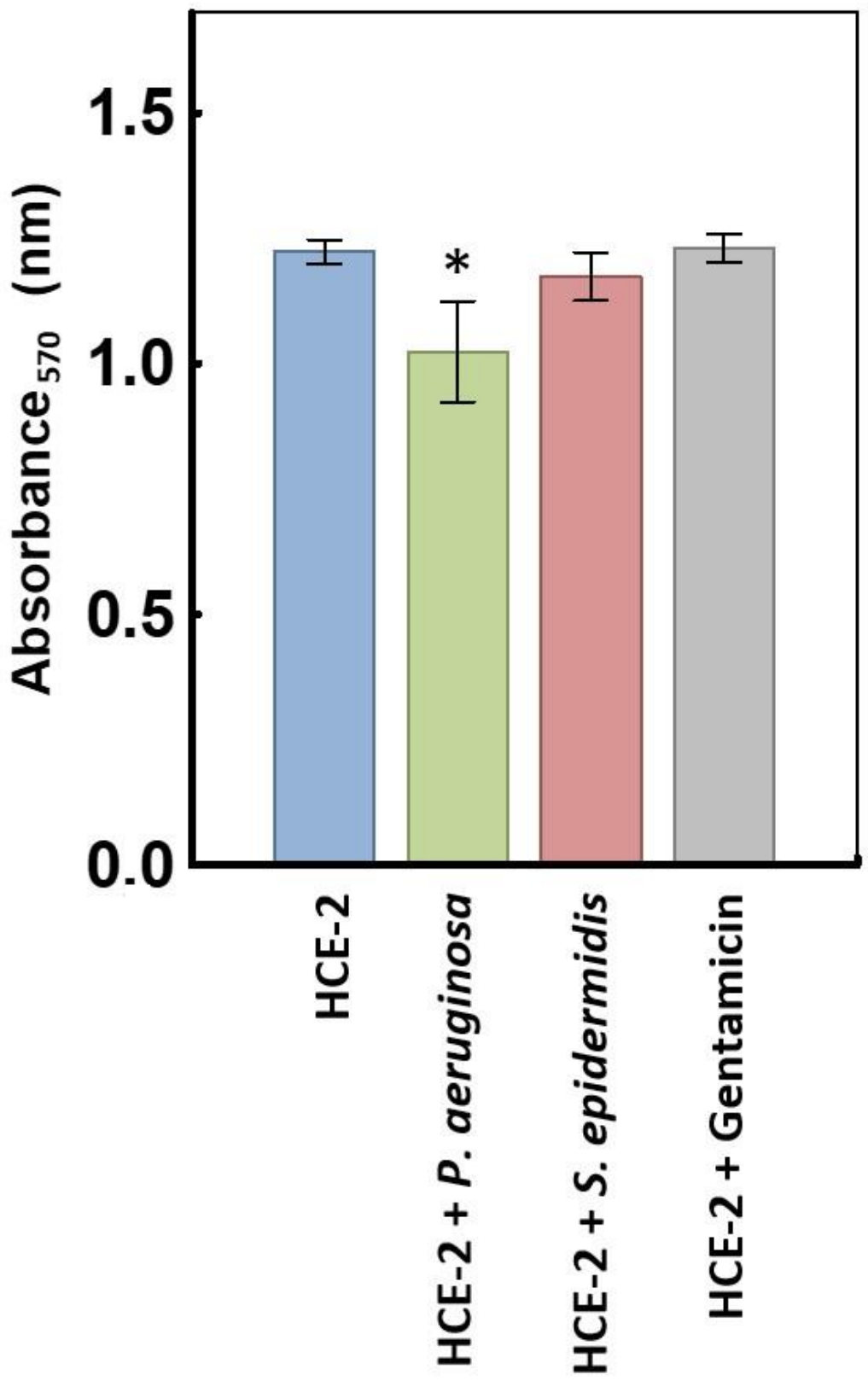

Supplementary Figure 9. Effect of gentamicin treatment on HCE-2 cell metabolic activity. Metabolic activity of HCE-2 cells was assessed by MTT assay following exposure to *Pseudomonas aeruginosa* co-culture (6.25 μg/mL gentamicin), *Staphylococcus epidermidis* co-culture (0.5 μg/mL gentamicin), or gentamicin alone (6.25 μg/mL). Gentamicin alone did not significantly affect epithelial metabolic activity compared with untreated controls, whereas co-culture with *P. aeruginosa* induced a significant reduction. Data are presented as mean ± SD from n = 5 independent biological replicates. Statistical significance was assessed using the Mann–Whitney U test. $p < 0.05$ (*).
